# A toolbox of genes, proteins, metabolites and promoters for improving drought tolerance in soybean includes the metabolite coumestrol and stomatal development genes

**DOI:** 10.1186/s12864-016-2420-0

**Published:** 2016-02-09

**Authors:** Prateek Tripathi, Roel C. Rabara, R. Neil Reese, Marissa A. Miller, Jai S. Rohila, Senthil Subramanian, Qingxi J. Shen, Dominique Morandi, Heike Bücking, Vladimir Shulaev, Paul J. Rushton

**Affiliations:** Department of Biology and Microbiology, South Dakota State University, Brookings, SD57007 USA; Texas A&M AgriLife Research and Extension Center, Dallas, TX 75252 USA; School of Life Sciences, University of Nevada, Las Vegas, 89154 USA; INRA, UMR 1347 Agroécologie, 17 rue Sully, BP 86510, 21065 Dijon, CEDEX France; Department of Biological Sciences, University of North Texas, Denton, TX 76203 USA; Current address, Molecular and Computational Biology, Dana & David Dornsife College of Letters, Arts and Sciences, University of Southern California, Los Angeles, CA 90089 USA; Current address: Texas A&M AgriLife Research and Extension Center, Dallas, TX 75252 USA; Current address, 22nd Century Group Inc., 9530 Main Street Clarence, New York, 14031 USA

## Abstract

**Background:**

The purpose of this project was to identify metabolites, proteins, genes, and promoters associated with water stress responses in soybean. A number of these may serve as new targets for the biotechnological improvement of drought responses in soybean (*Glycine max*).

**Results:**

We identified metabolites, proteins, and genes that are strongly up or down regulated during rapid water stress following removal from a hydroponics system. 163 metabolites showed significant changes during water stress in roots and 93 in leaves. The largest change was a root-specific 160-fold increase in the coumestan coumestrol making it a potential biomarker for drought and a promising target for improving drought responses. Previous reports suggest that coumestrol stimulates mycorrhizal colonization and under certain conditions mycorrhizal plants have improved drought tolerance. This suggests that coumestrol may be part of a call for help to the rhizobiome during stress. About 3,000 genes were strongly up-regulated by drought and we identified regulators such as ERF, MYB, NAC, bHLH, and WRKY transcription factors, receptor-like kinases, and calcium signaling components as potential targets for soybean improvement as well as the jasmonate and abscisic acid biosynthetic genes *JMT*, *LOX1*, and *ABA1*. Drought stressed soybean leaves show reduced mRNA levels of stomatal development genes including *FAMA*-like, *MUTE*-like and *SPEECHLESS*-like bHLH transcription factors and leaves formed after drought stress had a reduction in stomatal density of 22.34 % and stomatal index of 17.56 %. This suggests that reducing stomatal density may improve drought tolerance. MEME analyses suggest that ABRE (CACGT/CG), CRT/DRE (CCGAC) and a novel GTGCnTGC/G element play roles in transcriptional activation and these could form components of synthetic promoters to drive expression of transgenes. Using transformed hairy roots, we validated the increase in promoter activity of *GmWRKY17* and *GmWRKY67* during dehydration and after 20 μM ABA treatment.

**Conclusions:**

Our toolbox provides new targets and strategies for improving soybean drought tolerance and includes the coumestan coumestrol, transcription factors that regulate stomatal density, water stress-responsive WRKY gene promoters and a novel DNA element that appears to be enriched in water stress responsive promoters.

**Electronic supplementary material:**

The online version of this article (doi:10.1186/s12864-016-2420-0) contains supplementary material, which is available to authorized users.

## Background

Among the abiotic stresses, drought is the single greatest factor that limits global food production [1]. New targets for the potential improvement of drought responses in crop species are therefore valuable. Tolerance to drought is, however, a complex quantitative and multigenic trait that is largely controlled by small effect genes or QTLs [2–4]. There is also a significant environmental effect on water stress responses in plants and the genetic control of traits associated with tolerance to drought often shows low heritability. As a consequence, drought responses from hydroponics, growth chambers, greenhouses, and different field conditions vary. In addition, water stress in the field often occurs together with other abiotic stresses such as heat or high salinity, adding another layer of complexity. Under such field conditions, gene, protein and metabolite discovery becomes difficult because the whole system is constantly subjected to various degrees of different stresses in varying combinations.

Drought responses have previously been studied in soybean using both pot-based systems (PSys) and hydroponics systems (HSys) [5–7]. PSys are more similar to field conditions with a slower rate of water loss that allows acclimation to the stress [5]. On the other hand, the rapid stress associated with the removal of soybean plants from a hydroponics solution results in a more uniform response to the stress and this may facilitate gene, protein, and metabolite discovery. Expression profile analyses of both systems show that although there are differences, many genes appear to show similar expression characteristics, for example *GmaxADC2-like* and *GmaxADC2-like1* [5].

Soybean is an important crop and several transcriptome analyses of the response to drought have been reported [8, 9]. Chen et al. [8] reported a genome-wide transcriptional analysis of two soybean genotypes under dehydration and rehydration. They identified over one thousand differentially expressed genes (at least two fold change) and the genes primarily encoded transcription factors, protein kinases, and other regulatory proteins. Le et al. [9] used a PSys and studied soybean leaf tissue at late developmental stages under drought stress. They identified 6,500 differentially regulated genes (at least two fold change) with many upregulated genes encoding transcription factors, kinases, two-component systems or proteins with functions in abiotic stress such as late embryogenesis-abundant proteins. Neither of the two reports extended their observations beyond the transcriptome level. More recently, Shin et al. [10] studied transcriptomic changes due to water deficit in two soybean cultivars, one of which was a slow-wilting variety [10]. They found that transcriptional responses to water deficit in soybean involve not only known pathways such as down-regulation of photosynthesis but also up-regulation of processes such as protein transport and chromatin remodeling. The importance of roots and root architecture to soybean drought responses was illustrated in a recent article by Prince et al. [11]. Genetically diverse soybean germplasm lines were selected and lines 578477A and 088444 had higher later root number and forks in clay soil and a higher yield under water limitation. Similarly, in sandy soil, PI458020 had a thicker lateral root system and higher yield under water limitation [11].

Here, we use a HSys-based approach for systems level analyses and identify targets for the improvement of soybean drought tolerance. Previous analyses from soybean have not been as extensive as the data presented here that combines physiological, transcriptomic, proteomic, metabolomics, and promoter analyses from the same samples. We can therefore make direct comparisons between changes in the different levels of the system. We identified 2,972 genes that were differentially regulated in leaves and 1,394 in roots (≥8-fold). In the same samples, we identified 95 biochemicals that show a statisically significant change in level (*p* < 0.05) in leaves and 163 biochemicals that show changes in roots. We suggest a new drought tolerance mechanism in legumes linking drought, coumestrol and mycorrhiza. We propose that drought induces an increase in coumestrol in the roots. This promotes the growth of mycorrhizal fungi, improves water use efficiency, and thereby enhances plant tolerance to drought stress. We present a toolbox for improving soybean drought tolerance consisting of targets at the gene, protein, and metabolite levels together with promoters and promoter elements for expressing transgenes. Finally, we discuss new strategies using these tools for the improvement of drought tolerance in soybean.

## Results

Our aim was to produce a list of targets whose manipulation might lead to increased drought tolerance in soybean (What are we going to express?). In addition, we also sought to produce a set of tools for the expression of these transgenes (How are we going to express it?) These potential transgenes and promoters (both native and synthetic) make up a toolbox for new strategies to improve drought tolerance. The components of this toolbox are listed in Table 1 along with comparisons with similar targets from other systems.

### The physiological level

**Fig. 1:**
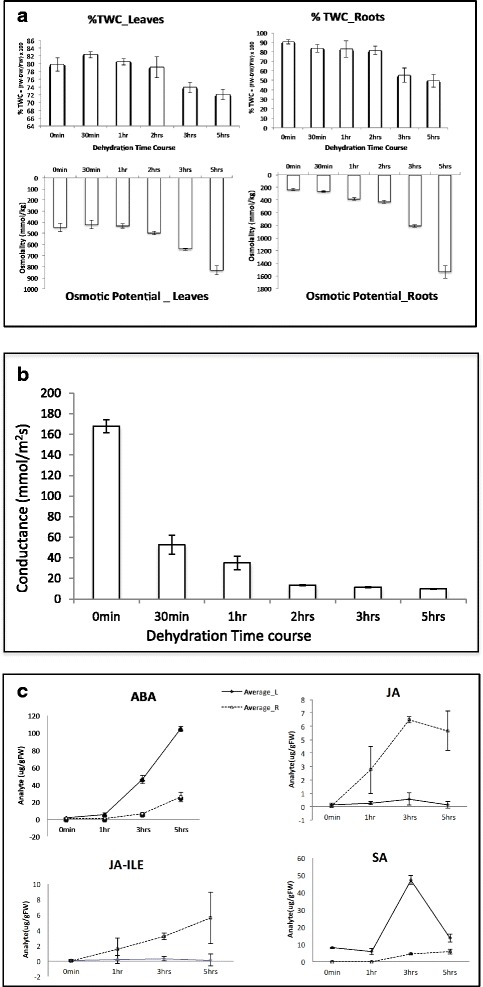
Physiological responses during water stress. **a** Total water content and osmotic potential. **b** Stomata conductance. **c** Phytohormone levels. Error bars show mean ± standard error for nine independent plants for each time point. Leaf is denoted by a solid line and root by a dotted one

In the field, it is common for plants to encounter abiotic stresses simultaneously. It is therefore difficult to characterize the signaling web that is associated with any one particular stress. Consequently, we performed experiments using hydroponic conditions where temperature, relative humidity, and the light regime were controlled. This minimized the effect of abiotic stresses other than water deficit. Soybean plants were subjected to a rapid and uniform water deficit stress by removing the plants from the hydroponics solution by means of the pots. In this way, wound responses were avoided by not touching the plants and harvesting was achieved quickly. A time course of five hours was chosen because five hour dehydrated plants were still able to recover and re-grow when put back into the hydroponics solution, showing that plant death had not occurred. To monitor the extent of the response to water stress physiological parameters were monitored. In roots, an 11 % decrease in total water content (%TWC) from 3–5 h of dehydration was observed while a 10 % decrease in leaves %TWC from 2–5 h was observed (Fig. 1). This observation is accordance with the similar trends seen in experiments performed in soil [12]. The osmotic potential showed a similar trend. In contrast, stomatal conductance revealed rapid stomatal closure within 30 min (Fig. 1). The stomatal conductance dropped to about one third of the control level by 30 min and after 2 h, the stomata were essentially closed.

In addition, the levels of the phytohormones abscisic acid (ABA), jasmonate (JA), jasmonate-isoleucine (JA-ILE), and salicylic acid (SA) were determimed. The most notable response was a rapid rise in JA and its biologically active conjugate JA-ILE in the roots (Fig. 1). In leaves there was a strong increase in ABA levels starting after one hour of drought. The increase in roots was less than in leaves (Fig. 1). This greater increase in ABA levels in leaves was mirrored by a 156-fold up-regulation in leaves and 32-fold induction in roots of the abscisic acid biosynthesis gene *ABA2* (*GLYMA11g18570*). ABA2 catalyzes the conversion of xanthoxin to abscisic aldehyde. Abscisic aldehyde is then converted to ABA. *ABA2* is therefore a candidate for the manipulation of soybean drought responses (Table 1). SA levels rose slightly in roots but showed a large spike in level in leaves between one and five hours. Cyanoalanine (an indicator of ethylene biosynthesis) was elevated at the earliest time-point in leaf tissue suggesting that ethylene also plays an early role in the response.

**Table 1:** Genes, proteins, metabolites, and promoters that are promising tools for the improvement of soybean drought responses

	Tissue	Observations	Comments
		Up-regulated genes	
Homeobox leucine zipper	Leaves and roots	In leaf about 100-fold induced after 2 h and 150-fold after five hours. In root about 20-fold after five hours. The two proteins are 90.2 % similar.	The most similar Arabidopsis orthologue is ATHB12. The homeodomain-leucine zipper (HD-Zip) class I transcription factors ATHB7 and ATHB12 modulate abscisic acid signalling by regulating protein phosphatase 2C and abscisic acid receptor gene activities.
*GLYMA16g02390*			
*GLYMA07g05800*			
ERF/AP2	Leaves and roots	The most strongly up-regulated transcription factor gene in leaves after two hours with an inducition of 135-fold. Induced 36-fold after one hour in roots.	The most similar Arabidopsis gene is the ERF transcription factor ABA REPRESSOR1 (ABR1). ABR1 is expressed in response to ABA, osmotic stress, sugar stress and drought. Mutants are hypersensitive to these stresses.
*GLYMA05g32040*			
ERF/AP2	Leaves and roots	The most strongly up-regulated transcription factor gene (573-fold after five hours in leaves). 10-fold induced in roots.	Another AP2/ERF transcription factor that is similar to Arabidopsis ABR1.
*GLYMA20g30840*			
ERF/AP2	Roots	The third most strongly induced soybean gene at early time points in root and the most strongly up-regulated AP2/ERF gene.	Our yeast 2-hybrid analyses show that GLYMA03g26310 interacts with the drought inducible WRKY transcription factor GmWRKY53. Similar to AtERF13 that is involved in regulating various biotic and abiotic stresses.
*GLYMA03g26310*			
ERF/AP2	Leaves	10-fold and 23-fold induced in leaves after five hours.	Our yeast 2-hybrid analyses show that both proteins interact with the drought inducible WRKY transcription factor GmWRKY53 [40].
*GLYMA10g33810*			
*GLYMA20g33800 (paralogs with 90.5 % identity)*			
			Member of the subfamily B-3 of ERF/AP2 transcription factors. B-3 includes ATERF-6 that acts as a central regulator of leaf growth under water-limiting conditions in Arabidopsis.
WRKY	Leaves	Rapid and transient induction in leaves. Maximum of 71-fold induction after 3 h.	Group IIe WRKY transcription factor.
*GLYMA01g43130 (GmWRKY161)*			
WRKY	Leaves	Transiently up-regulated in leaves with a maximum of 21-fold after two hours.	Similar to AtWRKY6 that is implicated in regulating senescence, defence responses, arsenate uptake, boron deficiency, and low phosphate responses.
*GLYMA17g04710 (GmWRKY112)*			
NAC	Leaves and roots	Strongly induced in leaves with a maximum of 148–fold after three hours. Also strongly induced in roots with a maximum of 70-fold after five hours.	Apparent orthologue of Arabidopsis RD26/ANAC071 RD26/ANAC071 is induced in response to desiccation. It is localized to the nucleus and acts as a transcriptional activator in ABA-mediated dehydration responses.
*GLYMA12g22880*			
NAC	Leaves and roots	Similar to GLYMA12g22880. Strongly induced in leaves with a maximum of 121–fold after three hours. Also strongly induced in roots with a maximum of 47-fold after five hours.	Similar to Arabidopsis RD26/ANAC071 and ANAC055 both of which appear to regulate stress responses.
*GLYMA12g35000*			
bHLH	Roots	The most strongly early up-regulated transcription factor in roots (50-fold after 30 min) and the second highest induced gene at this time point.	Similar to bHLH92 (At5g43650) that functions in plant responses to osmotic stresses.
*GLYMA12g22880*			
MYB	Leaves and roots	R2R3-MYB transcription factor 74-fold induced after three hours in leaves. 10-fold induced after five hours in roots.	Many MYB transcription factors regulate stress responses but the role of GLYMA05g35050 in unknown.
*GLYMA05g35050*			
C3H	Leaves and roots	Up-regulated in the root 80-fold after three hours and 15-fold in the leaves after three hours.	SOMNUS is a key negative regulator of seed germination that acts downstream of PHYTOCHROME INTERACTING FACTOR3-LIKE5 (PIL5). The role of SOMNUS outside of seed germination is unclear.
*GLYMA06g44440 (SOMNUS)*			
ERF/AP2	Leaves	Large induction of 427-fold after three hours in leaves. Not significantly induced in roots.	Similar to the tobacco proteins NtERF211 and NtERF204 both of which are strongly induced by drought.
*GLYMA10g36760*			
JAZ/TIFY	Leaves and roots	Rapidly up-regulated in roots with a later maximum of 61-fold induction after three hours. 18-fold induced in leaves after three hours.	TIFY5A-like transcription factor. Consistent with a role of JA in drought responses in soybean.
*GLYMA15g09980*			
LEA protein	Leaves and roots	The most strongly up-regulated gene in the soybean genome (1,018-fold in leaves after three hours). Also up-regulated to a lower level in roots.	LEA proteins are well known drought response genes. The massive induction of this particular LEA gene suggests it could be a useful target for soybean improvement.
*GLYMA10g07410*			
Glucose and ribitol dehydrogenase	Leaves and roots	One of only three genes induced over 1,000-fold by drought (1,009-fold after five hours in leaf). Also up-regulated to a lower level in roots.	May be involved in carbohydrate metabolism and the acquisition of desiccation tolerance (uniprot.org).
*GLYMA03g39870*			
Auxin efflux carrier	Roots	610-fold induced in leaves.	Role in drought responses is unknown.
*GLYMA11g09250*			
Cytokinin hydroxylase-like	Leaves and roots	Very rapid early induction and maximum of 60-fold after five hours. 8-fold induction in leaves.	This suggests that trans-hydroxylation is involved in the regulation of cytokinin metabolism and signaling.
*GLYMA10g37920*			Potential for improving drought responses unknown.
Jasmonic acid carboxyl methyltransferase (JMT)	Roots	Root-specific early induction and maximum of 237-fold after five hours	Catalyzes the formation of methyl jasmonate from jasmonic acid and is a key enzyme for jasmonate-regulated plant responses (Seo et al., 200).
*GLYMA16g24800*			
Lipoxygenase LOX1	Roots	Later root-specific induction with a maximum of 89 –fold after five hours.	Involved in the biosynthesis of JA
*GLYMA13g42320*			
ELI3-2 mannitol dehydrogenase	Roots	Strongly induced in both tissues with a maximum of 618-fold in leaves after five hours and 157 – fold in roots at the same time point.	Mannitol dehydrogenase (MTD) is a prime modulator of mannitol accumulation in plants (Zamski et al., 2001).
*GLYMA01g02580*			
*ABA2* (*ABA deficient 2*)	Leaves and roots	156-fold up-regulated in leaves after five hours and 32-fold induced in roots at the same time point.	ABA2 is an abscisic acid biosynthesis enzyme that belongs to a family of short-chain dehydrogenases/reductases. It is also called xanthoxin dehydrogenase. ABA2 catalyzes the conversion of xanthoxin to abscisic aldehyde. Abscisic aldehyde is then converted to ABA. *ABA2* is a good candidate for improvement of soybean drought responses.
*GLYMA11g18570*			
Protein phosphatase 2C (similar to AIP1, HIGHLY ABA-INDUCED PP2C GENE 2, HONSU)	Leaves and roots	72-fold up-regulated in leaveas and 31-fold in roots.	Similar to the *HIGHLY ABA-INDUCED PP2C GENE 2* of Arabidopsis that functions as positive regulator of ABA (Lim et al., 2012).
*GLYMA01g43460*			
		Down-regulated genes	
Stomatal Density and Distribution 1 (SDD1)	Leaves	mRNA level goes down 17-fold in leaves at the earliest time point. At this time point the eighth most strongly down-regulated gene.	Appears to be part of a long term response to drought that reduces the amount of stomata in new leaves.
*GLYMA19g35200*			
bHLH (Group 10 Ia)	Leaves	mRNA level goes down 31-fold in leaves after two hours. At this time point the tenth most strongly down-regulated gene.	Group 10 IA bHLH gene related to the regulators of stomatal development in Arabidopsis, FAMA, SPEECHLESS, and MUTE
*GLYMA06g35330*			
GUARD CELL HYDROGEN PEROXIDE-RESISTANT1 (GHR1)	Leaves	Within 30 min, the levels of mRNA encoding Glyma15g13840 and Glyma09g02881 fall to about one third of their non-stressed levels and reach a 7-9-fold reduction after 3–5 h.	GHR1 mediates ABA and hydrogen peroxide-regulated stomatal movement under drought stress [21] and GHR1 is a critical early component in ABA signaling.
*GLYMA15g13840* and *GLYMA09g02881*			
		Constitutive genes	
GmICHG	Roots	Among the 170 most highly expressed genes in soybean roots	The isoflavone conjugate-hydrolyzing β-glucosidase (GmICHG) may release conjugated coumestrol from its latent form in the vacuole to be excreted from the roots to promote plant-microbe interactions.
*GLYMA12g05770*			
		Metabolites	
Coumestrol	Roots	An isoflavanoid with a striking 161-fold increase after three hours in roots. Levels have increased 46-fold after just one hour.	Previous reports show that coumestrol stimulates mycorrhizal colonization and hyphal growth and under certain conditions mycorrhizal plants can have improved drought tolerance. Possible novel drought tolerance mechanism where drought induces an increase in coumestrol, increased interactions with mycorrhiza and thereby enhances tolerance to drought stress. Coumestrol is therefore a potential biomarker for water stress and a promising target for legume improvement.
Formononetin	Roots	An isoflavanoid that increases almost 10-fold in roots.	Like coumestrol, formononetin may be involved in signaling to the rhizosphere as a response to drought.
Allantoin	Leaves and roots	Allantoin levels increase nearly 8-fold in leaves and 4.2-fold in roots.	The purine metabolite allantoin enhances abiotic stress tolerance through synergistic activation of abscisic acid metabolism [17]. Mutants that accumulate more allantoin show enhanced tolerance to drought.
Raffinose	Leaves and roots	In the roots there is a large increase in raffinose after three and five hours, reaching a peak of 12.89-fold increase after five hours of drought. A similar rise in leaves reaches 21.8-fold after five hours.	The raffinose pathway can provide osmolytes in situations of low water potential.
Galactinol	Leaves	In the leaf, galactinol increases 9.6-fold but there is no significant increase in roots.	Galactinol acts as an osmolyte in situations of low water potential.
γ-aminobutyrate (GABA)	Roots	GABA levels increase 13-fold in roots.	The GABA shunt is a stress response pathway, the functions of which include controlling cytoplasmic pH, maintaining C/N balance by converting glutamate in the cytosol to succinate in the TCA cycle, and aiding in oxidative stress protection by generating NADH and succinate.
		Hormones	
ABA (abscisate/abscisic acid)	Leaves and roots	The ABA concentration increased 7.8-fold after five hours in leaf tissue and appears to increase over 5-fold in roots. Strong *ABA2* up-regulation is consistent with increasing ABA levels. Many ABA responsive genes are up-regulated in both tissues. Components of ABA signaling such as protein phosphatase 2C genes are also up-regulated.	ABA plays a central role in regulating drought responses in soybean.
JA (Jasmonate)	Roots	There was a rapid rise in JA and its biologically active conjugate JA-ILE in the roots All of the biosynthetic enzyme genes in the JA biosynthetic pathway are rapidly and coordinately up-regulated in roots. Many JA signaling components such as JAZ repressors are differentially regulated.	JA clearly plays an important role in the response to drought in soybean. Lipoxygenase, allene oxidase synthase, allene oxidase cyclase, and 12-Oxo-PDA-reductase genes all show induction in roots and may be good targets for improvement of soybean.
Ethylene (Ethene)	Leaves and roots	Cyanoalanine (an indicator of ethylene biosynthesis) was elevated at the earliest time-point in leaf tissue suggesting that ethylene plays an early role in the response. The biosynthetic enzyme genes in the ethylene biosynthetic pathway show up-regulation.	Ethylene plays a role in the regulation of drought responses.
		Proteins	
MAP kinase 2-like	Leaves	Increases 3.63-fold at the protein level.	Similar proteins in *Medicago truncatula* and Arabidopsis respond to many different stress stimuli.
*GLYMA05g37480*			
Inositol-polyphosphate 5-phosphatase	Leaves	Increases 3.63-fold at the protein level.	A similar Arabidopsis protein (AT1G05630) is induced in response to ABA and wounding treatments.
*GLYMA07g40360*			
		Promoters	
ABRE CACGT/CG	Leaves and roots	The well-characterized ABA Response Element is found in the promoters of many of the most strongly up-regulated genes and the ABREs are clustered in the first 250–500 bp of the promoters.	The ABRE is a binding site for certain members of the bHLH and bZIP transcription factor families. Synthetic promoters containing ABREs or ABREs in combination with other drought responsive elements may prove useful for driving transgenes in projects aimed at improving drought responses.
CRT/DRE motif CAC/TCGACC	Leaves and roots	Found in ten of the root early up-regulated promoters	The Cold/Dehydration Responsive Element is the binding site for AP2/ERF transcription factors. Given that many ERF genes are strongly up-regulated by drought and that several are listed in this table as potential targets for improving drought responses then their potential binding sites are excellent candidates for building blocks for synthetic drought-inducible promoters.
GTGCnTGC/G Element	Leaves	Found by MEME in found in sixteen of the leaf late up-regulated promoters	Novel potential element. Will require detailed functional characterization and identification of cognate transcription factors.
*GmWRKY71 and GmWRKY67 promoters*	Roots	Drought inducible. The *GmWRKY17* promoter is also responsive to ABA.	Drought and cold inducible promoters.
*GmWRKY53 and GmWRKY112 promoters*	Roots and leaves	Drought inducible. *GmWRKY53* and −*112* promoters respond positively to water stress through exogenous application of salt and PEG.	Drought and salt inducible promoters.

### The metabolome level

To determine metabolite responses samples were analyzed by liquid chromatography/mass spectrometry (LC/MS, LC/MS2) and gas chromatography/mass spectrometry (GC/MS) platforms. 207 biochemicals were detected in the root tissue and 241 in leaf tissue. Changes in the biochemical profile of root were far more extensive than those observed in leaf (Additional file 1: Table S1). The changes were also faster because statistically significant changes were only observed in leaf after 120 min. This is similar to the transcriptome data in leaf where there were no significant changes at the earliest two time points (see below).

Previous studies have shown that sugars (such as raffinose family oligosaccharides, sucrose, trehalose and sorbitol), sugar alcohols, amino acids, and amines accumulate under drought stress [13]. These function as osmolytes because they can accumulate to high concentrations within cells without impairing cellular function [14]. Starting at one hour, an increase in many sugars was observed in roots including trehalose, raffinose, mannitol, pinitol, sucrose and kestose. In leaves, trehalose was not detected and pinitol did not increase. In both roots and leaves, the most predominant accumulated sugars were raffinose and galactinol (Additional file 2: Figure S1).

**Fig. 2:**
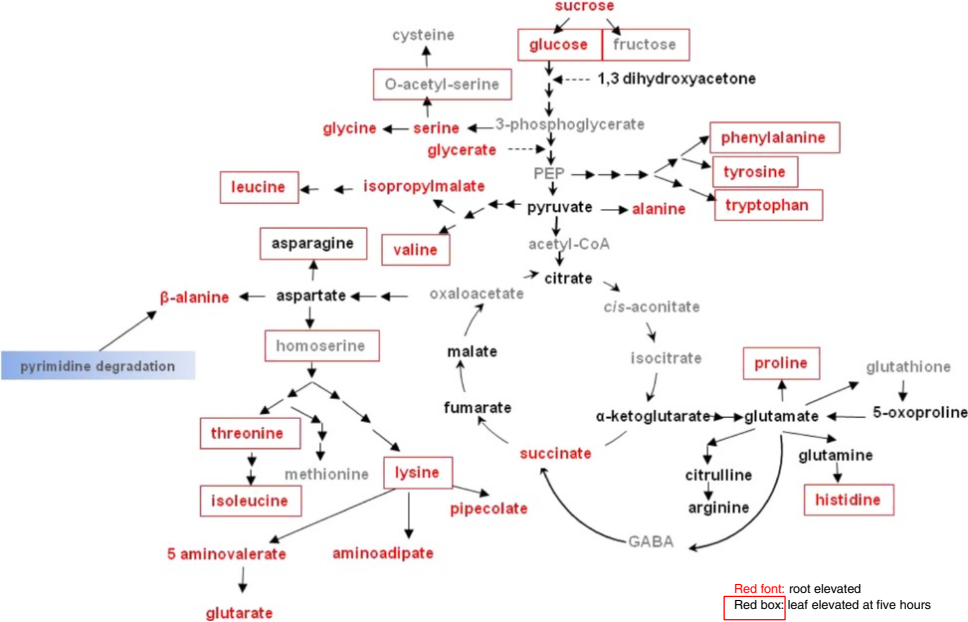
Amino acids elevated by water stress. Red font indicates significantly root elevated and the red boxes leaf elevated

Amino acids can also act as compatible solutes or osmolytes. Both leaves and roots accumulated various amino acids but increases were greater and induction faster in roots than in leaf material. In roots there was a steady increase in most amino acids from the 30 min time point to five hours. In contrast, in leaf only lysine and alanine were higher after 30 min and 1 h although most amino acids were higher by 5 h (Fig. 2). In roots, glycine, histidine, isoleucine, leucine, and valine all increased at least 10 fold.

Ammonia detoxification appears to be occurring and the conversion of ammonia into non-toxic forms appears critical in maintaining normal cellular functions during water stress [15, 16]. One early response was the accumulation of asparagine, allantoin and glutamine (Additional file 3: Figure S2). Asparagine and allantoin are the main metabolites responsible for nitrogen storage and transport. Glutamine is produced by the initial assimilation of ammonia by the action of glutamine synthetase. Recently, it has been demonstrated that the purine metabolite allantoin enhances abiotic stress tolerance through synergistic activation of abscisic acid metabolism [17]. Mutants that accumulate more allantoin show enhanced tolerance to drought. In our experiments, allantoin levels increased nearly 8-fold in leaves and 4.2-fold in roots. This identifies allantoin as a potential target for the improvement of soybean (Additional file 1: Table S1).

#### Coumestrol and a possible drought tolerance mechanism

**Fig. 3:**
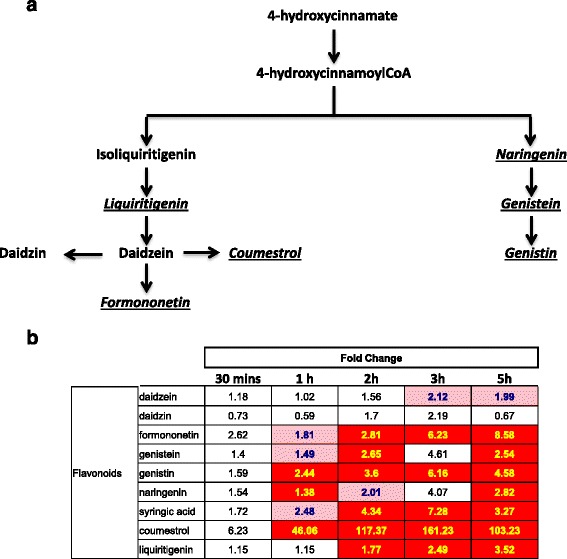
Isoflavonoid biosynthesis in roots during drought. **a** The biosynthetic pathway leading to coumestrol, formononetin, and genistin. Underlined and italicized indicates statistically significant increases in concentration. **b** Heat map of statistically significant changes in isoflavonoids. Red cells indicate *p* ≤ 0.05 with the mean values significantly higher than the control. Light red cells indicate 0.05 < *p* < 0.10

The most dramatic observation at the metabolite level was a tissue-specific accumulation of various isoflavonoids in roots (Fig. 3). The greatest induction of any detected compound was seen with coumestrol, with a striking 160-fold increase after three hours (Fig. 2 and Additional file 1: Table S1). Several isoflavones, such as daidzein and formononetin, have been reported to play roles in signaling and communication in rhizosphere plant-microbe interactions [18, 19]. In our study, daidzein shows only a two fold increase whereas formononetin levels increase about 8.5-fold. The intermediate for both formononetin and coumestrol, daidzein, does not increase suggesting that the major flux in soybean roots during drought is through the pathway that leads to coumestrol with less flux through the pathway that leads to formononetin. Our results therefore suggest a link between drought and coumestrol in legumes. Drought induces a large increase in coumestrol in the roots. We hypothesize that this increase promotes the growth of mycorrhizal fungi and thereby enhances plant tolerance to drought stress. Coumestrol is therefore a potential biomarker for drought and a promising target for legume improvement (Table 1).

### The transcriptome level

To find targets at the mRNA level for improving drought tolerance genome-wide transcriptome profiles were generated using a custom designed oligoarray containing probes for all gene models from v1.0 of the soybean genome. Three biological replicates were used. Our strategy of eliminating stresses except water deficit was validated by the large number of genes that showed high levels of differential expression. This likely reflects a lack of stress in the control plants coupled with a uniform response of the tissues. To concentrate on the mostly highly induced or repressed genes, we set a threshold of ≥ 8-fold for differentially regulated genes. Even with this high threshold 2,972 genes were differentially expressed in leaves and 1,394 in roots. A complete list of all differentially expressed genes is presented in Additional file 4: Table S2.

Changes in mRNA levels occured more rapidly in the root than the leaves. At the earliest time-point in roots (30 min), 128 genes showed at least 8-fold induction. Using Singular Enrichment Analysis, the most significant early GO terms were transcription factor activity and transcription regulator activity (Additional file 5: Figure S3 and Additional file 6: Table S3). By five hours 1195 genes were differentially expressed and the most significant GO terms now also include downstream target gene activation (Additional file 6: Table S3, Additional file 7: Table S4, Additional file 8: Table S5).

In contrast to roots, there were no significant changes in the transcriptome in leaf in the first two hours. This is in agreement with the metabolomics data that show that changes in the biochemical profile of root tissues were far more extensive and more rapid than that observed in leaf tissues. By two hours, however, 640 genes were differentially expressed (Additional file 4: Table S2 and Additional file 5: Figure S3) and after five hours, it was clear that major transcriptional re-programming was occurring because this number had increased to 2,652, representing about 4.7 % of total genes (Additional file 9: Table S6, Additional file 10: Table S7). The changes between two hours and five hours again illustrated a progression from signaling to downstream responses aimed at protecting the plant against drought.

**Fig. 4:**
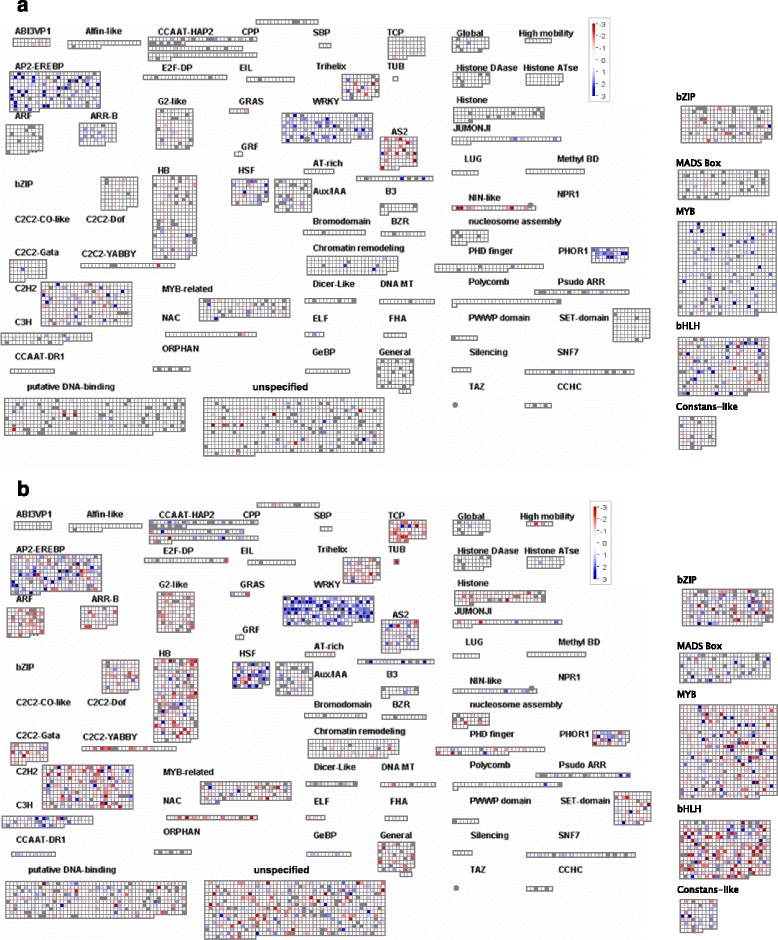
Visualization of differentially expressed TF genes with MapMan. **a** Roots after 30 min of drought. **b** Leaves after two hours of drought. The figure was constructed using log2-transformed ratios of induced versus control. The scale bar is a log2 scale and the most intense colors represent 8-fold change. A complete analysis of all time points is presented in Figure S1. Blue denotes increase and red decrease. Each square is a gene model

One major focus of our analyses was differentially expressed transcription factors because transcription factors are good candidates for improving drought tolerance. Major differences were observed between leaves and roots in both the timing and nature of the transcription factor genes that were differentially expressed. In roots, after 30 min, 44 of the 134 differentially regulated genes encoded transcription factors (Additional file 11: Table S8). Transcription factor gene expression in leaves (Additional file 12: Table S9) is qualitatively different from early time-points in the root. MapMan analysis suggests that ERF, WRKY, HSF, MYB, and bHLHs are the major families of up-regulated transcription factors (Fig. 4). Taken together, we see major differences between leaves and roots in both the timing and nature of the transcription factor genes that are regulated at the mRNA level. These genes represent good targets for soybean improvement (Table 1).

Several classes of genes encoding other signaling molecules show differential regulation in either leaves or roots (Additional file 4: Table S2). These data suggest that protein kinases, protein phosphatase 2Cs, F-box family proteins, and ubiquitin protein ligases all play roles. Both GO and MapMan analyses also confirmed a role for the hormones ABA, SA, and ethylene (Additional file 6: Tables S3, Additional file 7: Tables S4, Additional file 8: Tables S5, Additional file 9: Tables S6, Additional file 10: Tables S7), consistent with their observed increases. In leaves, GO analyses (Additional file 9: Table S6 and Additional file 13: Table S10) also suggest a role for calcium signaling.

**Fig. 5:**
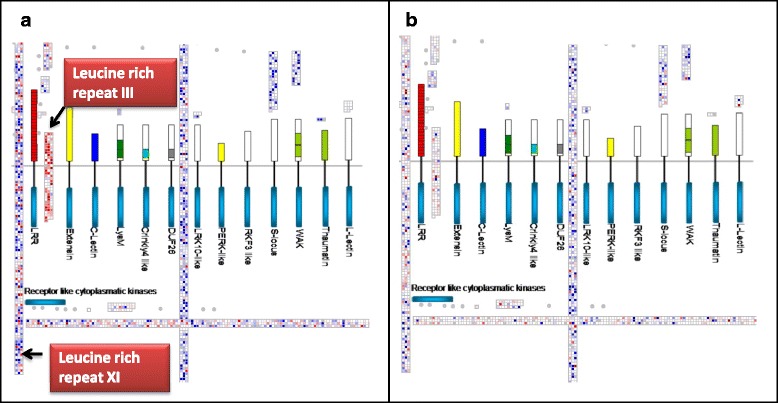
Visualization of differentially expressed receptor-like kinase genes with MapMan. The receptor kinase bin 30.2 is illustrated. **a** Leaf and (**b**) Roots. Subfamilies III and XI are indicated. The figure was constructed using log2-transformed ratios of induced versus control. The most intense colors represent 8-fold change. Blue denotes increase and red decrease

Receptor-like kinases (RLKs) are important components of early signaling including drought [20]. Analysis of RLK genes reveals a marked difference between root and leaf tissue (Fig. 5). Both tissues differentially regulate many RLK genes but leaf tissues show a striking down-regulation of about half of the LRR subfamily III genes (Fig. 5). Among these are *Glyma15g13840* and *Glyma09g02881*, two soybean orthologues of the Arabidopsis *GUARD CELL HYDROGEN PEROXIDE-RESISTANT1* (*GHR1*) gene. GHR1 mediates ABA and hydrogen peroxide-regulated stomatal movement under drought stress [21] and GHR1 is a critical early component in ABA signaling. Within 30 min, the levels of mRNA encoding Glyma15g13840 and Glyma09g02881 fall to about one third of their non-stressed levels and reach a 7-9-fold reduction after 3–5 h (Additional file 4: Table S2). This parallels a similar rapid drop in stomatal conductance. These data identify the soybean orthologs of *GHR1* as potential targets for improving drought tolerance via their effect on stomatal movement.

At the later time-points, downstream genes encoding proteins that protect the cell from the effects of water deficit showed increasing induction. These include water channel proteins, membrane transporters, proteins that protect and stabilize cell structures from damage by reactive oxygen species (detoxification enzymes such as glutathione S-transferase) and proteins that protect macromolecules (LEA, osmotin, chaperons) (Table 1 and Additional file 4: Table S2).

#### Water stress induced changes in the stomatal development program

**Fig. 6:**
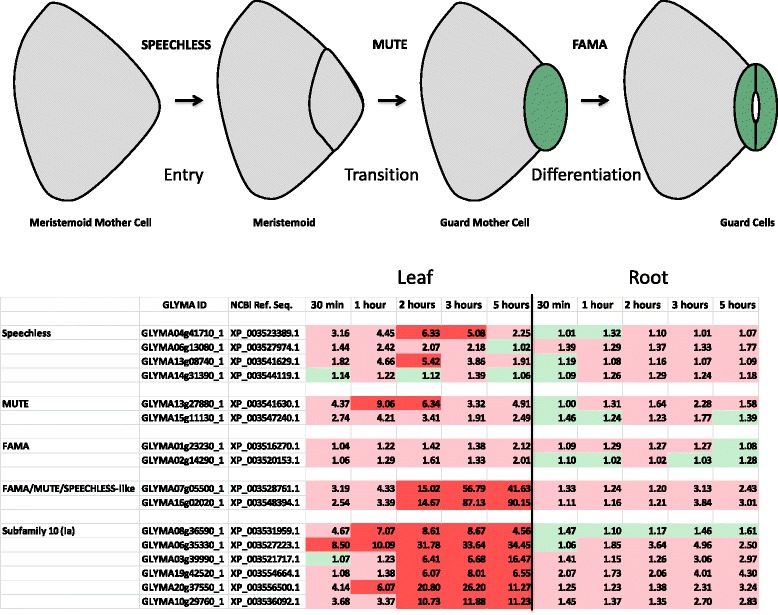
Stomatal development genes are down-regulated by drought. **a** Cartoon of the development of stomata and the role of SPEECHLESS, MUTE, and FAMA bHLH transcription factors. **b** Fold repression (red/pink) or activation (green) of stomatal development genes at the mRNA level in soybean during drought. Red denotes at least 5-fold change

The soybean bHLH transcription factor family contains 38 members that were strongly up- or down-regulated by drought and this response was markedly tissue specific (Additional file 14: Table S11). In roots the majority of genes were up-regulated. Interestingly, the picture in leaves was the opposite, with 18 out of 23 genes showing a reduction in mRNA to less than 12.5 % of unstressed levels (Fig. 6). A combined phylogenetic tree of the soybean and Arabidopsis bHLH gene families revealed that there are two major clusters of leaf down-regulated bHLH genes in soybean (Additional file 15: Figure S4). Subfamily 10 (Ia) contains eight down-regulated genes and subfamily 3 (IVa) contains 6 genes. Interestingly, subfamily 10 (Ia) bHLH includes three regulators of stomatal development in Arabidopsis, FAMA, SPEECHLESS, and MUTE [22]. All three transcription factors are positive regulators of stomatal development. This prompted us to look at the soybean orthologues of these genes.

**Fig. 7:**
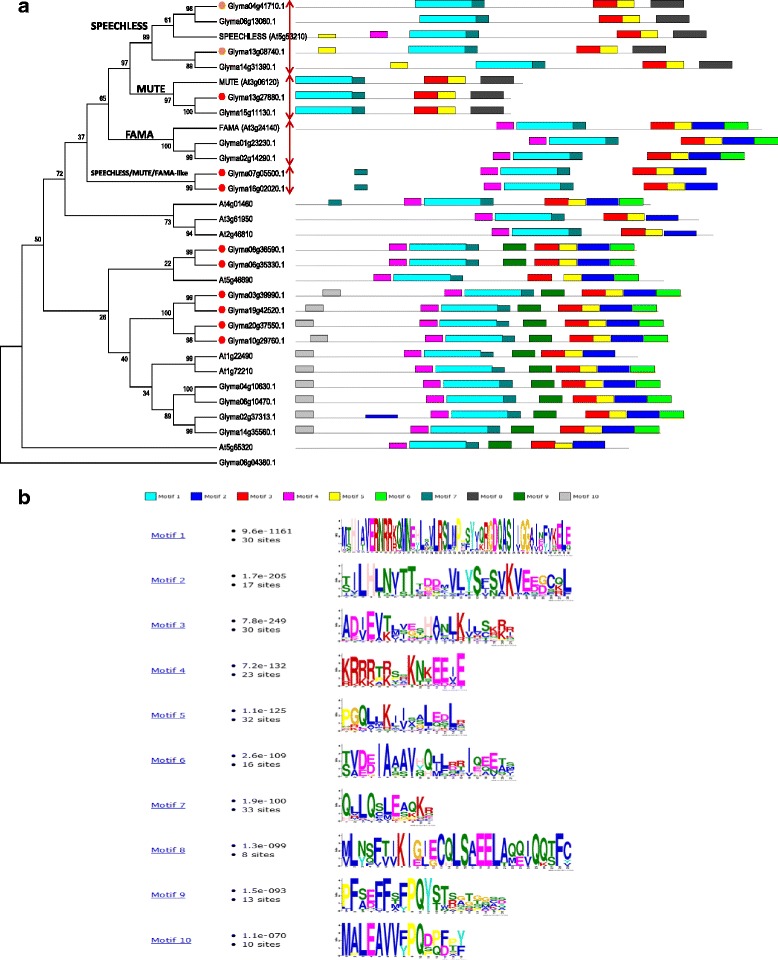
Phylogenetic analyses of the Group 10 (Ia) bHLH subfamily from soybean and Arabidopsis. **a** Neighbor Joining phylogenetic tree derived from a MUSCLE alignment of the full length proteins. SPEECHLESS, MUTE and FAMA-like genes are indicated. Numbers indicate bootstrap values from 1,000 replicates. Red dots denote 8-fold down-regulation and orange dots 5-fold. To the right of each protein is a cartoon of the protein architecture derived by MEME analysis. **b** The conserved protein motifs produced by MEME analysis

The soybean genome is a partially diploidized tetraploid [23] and therefore two soybean co-orthologs of *FAMA*, *SPEECHLESS*, and *MUTE* might be expected. The situation in soybean is, however, rather more complex. The three Arabidopsis bHLH transcription factors are closely related and form a clade with ten soybean bHLHs. There appear to be four co-orthologs of *SPEECHLESS* and two show reduction in mRNA levels of between 5- and 6.3 fold (Fig. 7). Soybean contains two co-orthologs of *MUTE* and one of the two *MUTE*-like genes shows a 9-fold drop in mRNA level. Interestingly, the two *FAMA*-like genes in soybean show no significant change in mRNA level. However, there are two additional soybean *FAMA*/*SPEECHLESS*/*MUTE*-like genes in the clade and these two genes are among the fifteen most strongly down-regulated soybean genes after three hours in leaves (out of over 50,000 expressed genes) with mRNA levels 87-fold (*Glyma16g02020*) and 56-fold (*Glyma07g05500*) less than unstressed plants (Fig. 7). Taken together, our results suggest that all three steps in the pathway that leads to the differentiation of stomata are down-regulated as a long-term response of soybean to lack of water however, the numbers and identities of the bHLH genes involved are different in legumes compared to the Brassicaceae.

FAMA, SPEECHLESS and MUTE regulate the last steps in the stomatal development signaling pathway (Fig. 6) but upstream components of the pathway are also known. In total 58 putative soybean orthologs of Arabidopsis stomatal development genes were identified and 24 of these showed differential expression (at least 5-fold change in mRNA level) (Additional file 16: Table S12). Strikingly, only one gene was up-regulated and only two genes showed significant variations in mRNA levels in root. STOMAGEN is an intracellular signaling peptide that is a positive regulator of stomatal patterning and a striking reduction of over 40-fold in the mRNA level of the *STOMAGEN*-like gene *Glyma08g45890* was observed. These data reveal that orthologues of genes that regulate stomatal development are among the most strongly down-regulated soybean genes during drought. This suggests that differentiation of stomata is reduced as a long-term response of soybean to drought. We validated the expression changes of fourteen genes from the oligo array using qRT-PCR (Additional file 17: Table S13). This included several stomatal development genes including *GLYMA16g02020* (FAMA-like), *GLYMA11g02520* (YODA MAP Kinase Kinase Kinase-like), and *GLYMA19g35200* (Stomatal Density and Distribution-1).

To establish directly whether drought does indeed cause a reduction in stomatal density, soybean plants were grown in soil for four weeks and well-watered. Half of the plants were then subjected to drought stress by withholding water for three weeks. After this period, all plants were well watered for three weeks. Leaves were selected from the youngest trifoliates that had formed after the imposed drought period. This was determined by marking the youngest trifoliates before ending the drought. The drought stressed plants had an average stomatal density of 22.34 % less than the well-watered plants and a reduction in leaf stomatal index of 17.56 % (Table 2). This suggests that using stomatal development genes to reduce the amount of stomata may be a good strategy to improve drought tolerance.

**Table 2:** Stomatal density and stomatal index in leaves formed before and after drought

	Average Stomatal Density per unit surface area	Leaf Stomatal Index [s / (s + c)]
Drought	46.08	16.11
Non-Drought	59.33	19.55
Percent Difference	−22.34	−17.56
*P*-Value	3.387 E-14	0.0001

### The promoter level

**Fig. 8:**
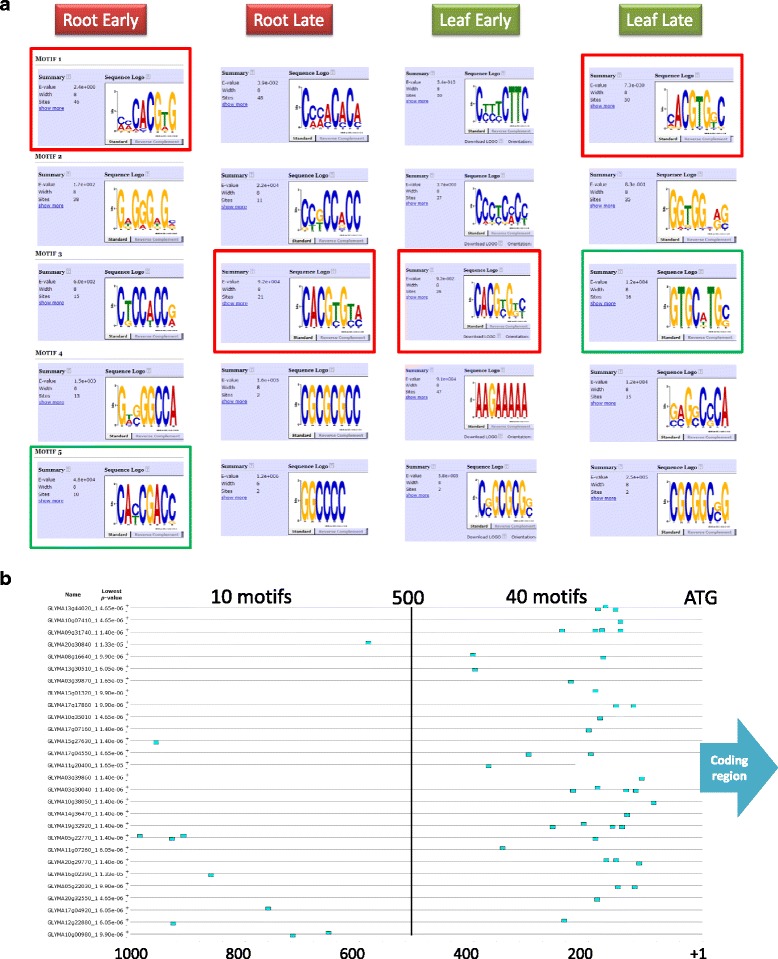
Non-random clustering of ABRE-like sequences in the 500 bp upstream of the ATG in the promoters of the fifty most strongly up-regulated genes at two time points from leaf and root. **a** The consensus sequences of the ABRE-like sequences derived from MEME for the four timepoints are shown, together with the number of sites and percentage of genes containing at least one site. **b** Cartoon representation of the ABRE-like sequences in the promoter regions of the genes from the leaf two hour time point. Blue rectangles denote ABRE-like sequences. Green rectangles denote non-ABRE sequences

Many approaches to the improvement of drought responses in soybean will involve the use of transgenes. The success or failure of these strategies may ultimately rest on the choice of promoter to regulate the expression of these transgenes. A data set was therefore constructed containing 1,000 bp of promoter region from the fifty most strongly induced genes at an early time-point (1 h root and 2 h leaf) and a late time-point (5 h in both). These were analyzed by MEME [24] for the presence of conserved sequence motifs that might serve as components of synthetic drought-inducible promoters for the controlled expression of transgenes. As expected, in all four data sets there was a striking occurrence of the G box-related ABRE sequence motif CACGT/CG (Fig. 8) with 42–60 % of the promoters containing at least one ABRE-like sequence. Notably, the positions of the ABRE-like sequences are non-random with the majority occurring within 500 bp of the predicted ATG. In leaf after two hours, 80 % of ABRE-like sequences were within the 500 bp closest to the ATG (Fig. 8) and 70 % were found in the first 280 bp. This is in contrast to most other sequences detected by MEME that showed a random distribution (data not shown). MEME analyses also identified the CRT/DRE motif CAC/TCGACC that was found in ten of the root early up-regulated promoters. In addition, the sequence motif GTGCnTGC/G (C/GCAnGCAC) was found in sixteen of the leaf late up-regulated promoters. This element would appear to be novel as it bears no significant similarity to any promoter element in the current list of binding sites in the AtcisDB database. It is unclear which transcription factors bind to the GTGCnTGC/G motif and it remains to be functionally characterized but it could prove to be a useful building block for synthetic drought-inducible promoters.

**Fig. 9:**
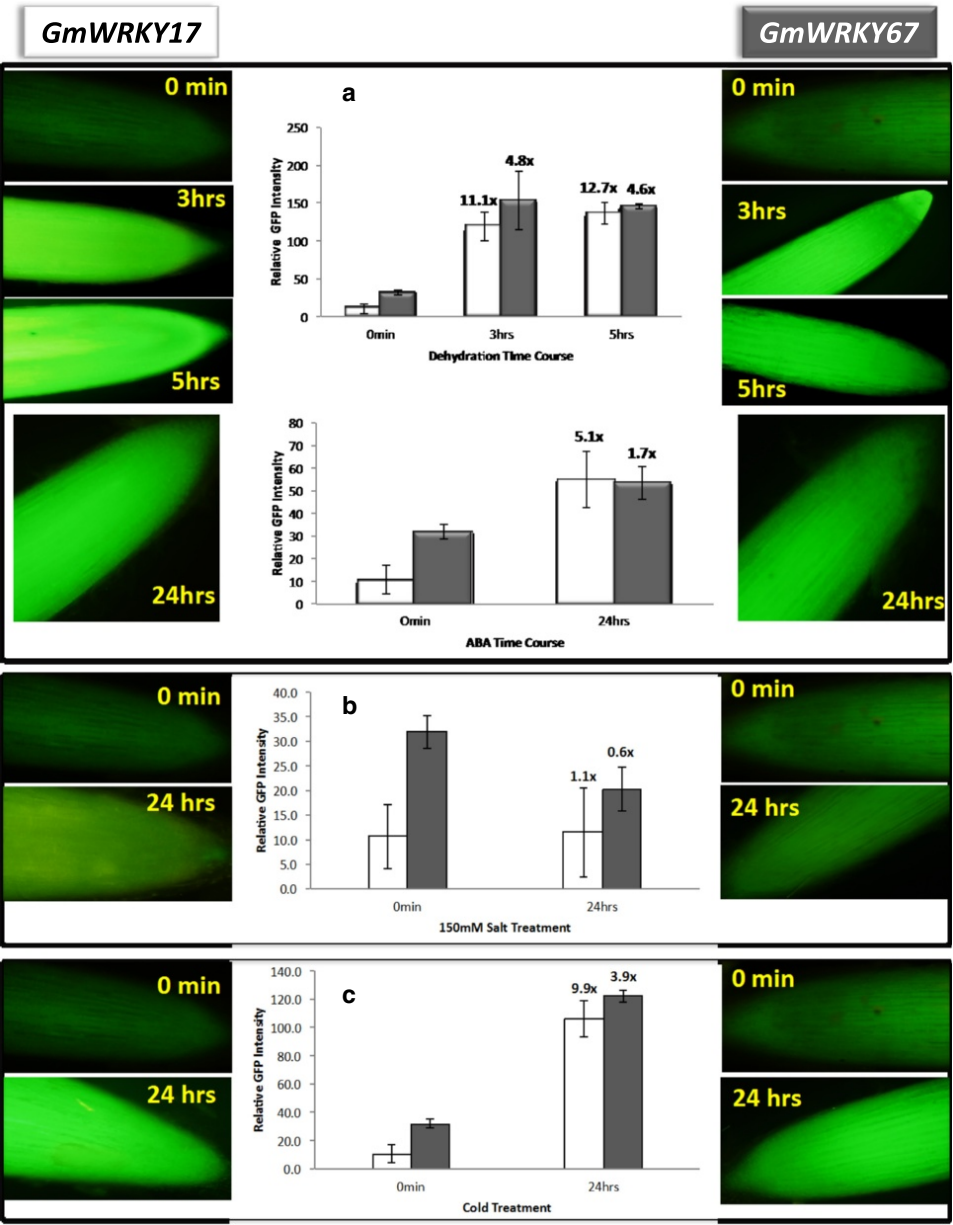
Validation of promoter activity of *GmWRKY17* and *GmWRKY67* in soybean hairy-roots during dehydration and 20 μM ABA treatment. Validation of promoter activity via visualization and quantification of a promoter:GFP construct of *GmWRKY17* and *GmWRKY67* in soybean hairy-roots during (**a**) dehydration (**b**) 20 μM ABA treatment and (**c**) cold. The time points in hours are shown. The graph shows mean ± standard error for 9 independent plants for each time point. The fold inducibilities are indicated

Our previous work in tobacco has shown that several WRKY gene promoters (notably *NtWRKY69*) direct water stress-inducible expression as shown by promoter:GFP or promoter:GUS analyses [16]. We therefore sought to validate and further characterize the inducibility of WRKY promoters from soybean because they could be good candidates for driving transgenes [25, 26]. Promoter:GFP constructs of two promising WRKY genes, *GmWRKY17* (GLYMA06g06530) and *GmWRKY67* (GLYMA13g44730) were transformed into soybean roots via hairy-root transformation and transgenic roots were subjected to drought. GFP quantification confirmed drought inducibility and revealed that the promoter of *GmWRKY17* directed 12.7-fold inducible expression and the *GmWRKY67* promoter 4.8-fold (Fig. 9). Additionally, the *GmWRKY17* promoter responded to ABA (Fig. 9) and both promoters were responsive to cold. This suggests that the promoters from *GmWRKY17* and *GmWRKY67* may prove useful for driving transgenes in projects aimed at improving drought responses. We have previously shown that the *GmWRKY53* and *GmWRKY112* promoters respond positively to exogenous application of salt and PEG [25, 26].

### Responses at the proteome level

Surprisingly, drought responses at the protein level have not been investigated extensively in soybean [27] and the few reports do not look at the metabolite and mRNA levels in the same samples. We therefore performed a proteomics study using the same set of root samples used for transcriptomics and metabolomics. A gel-free shotgun proteomics approach was employed that utilized Multi-Dimensional Protein Identification Technology (MuDPIT). Out of 2,471 identified proteins, 122 proteins were found to have significant differences in level after three hours or five hours compared to control roots (Additional file 18: Table S14). Strikingly, more proteins showed a reduction in abundance than an increase, suggesting that protein degradation/turnover is a characteristic of the drought response. Recently, the proteome of soybean roots subjected to short-term drought stress was studied [28]. Although only 28 proteins were identified that showed variations in abundance 21 of these showed a similar reduction in level to our observations.

Several trends could be observed (Additional file 18: Table S14). Firstly, metabolism-related proteins that are involved in energy production are reduced in abundance. This includes proteins involved in glycolysis, the TCA cycle, and oxidative phosphorylation. This correlates with a reduction in many photosynthesis-related genes at the mRNA level and shows that drought adversely affects photosynthesis and energy production and consequently reduces plant growth. Secondly, some signaling proteins were up-regulated at the protein level. This included a MAP kinase, casein kinase, receptor kinase, inositol 1,4,5-trisphosphate 5-phosphatase, and calmodulin-binding protein. Some are similar to stress-inducible genes/proteins from other plants (Table 1).

## Discussion

### New strategies for improving soybean drought responses

**Fig. 10:**
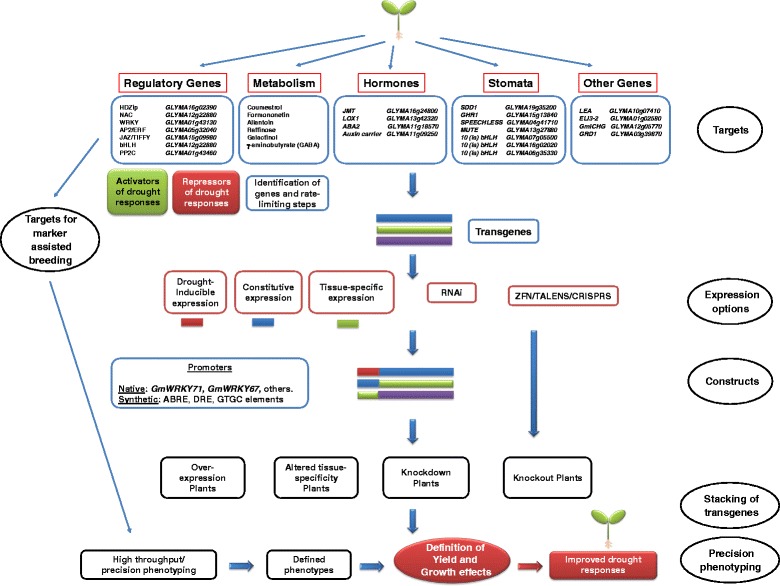
Strategies for improving soybean drought tolerance using the toolbox of genes, proteins, metabolites and promoters

We have analyzed soybean plants using systems biology approaches during water stress. Our extensive data sets revealed a number of novel biological insights and also potential transgenes, drought inducible promoters, and metabolic pathways to target in projects aimed at improving drought tolerance (Table 1). Figure 10 shows an overview of potential strategies to use this toolbox to produce soybean plants with improved drought tolerance. This includes overexpression, knockdown, knockout, and altered tissue-specific expression using specific regulatory genes, stomatal development genes, hormone biosynthesis and response genes, genes involved in secondary metabolism, and other downstream drought-inducible genes. Many of these genes could also form the basis of non-transgenic approaches using marker assisted breeding. In addition, the large increase in coumestrol observed in roots could make it a biomarker for drought because coumestrol levels can be easily measured and the increase in levels is both early and massive.

The first two parts of any strategy aimed at improving drought tolerance by transgenic means needs to answer two questions: What are we going to express? (the choice of transgene) and how are we going to express it? (the choice of promoter/expression cassette). It is likely that many previous projects have failed not because of a poor choice of transgene but rather due to the choice of an inappropriate promoter. Ectopic overexpression using promoters such as the CaMV 35S promoter have often been previously used and this uncontrolled expression may lead to improved drought tolerance but in many cases may also lead to reductions in yield due to constitutive activation of abiotic stress responses. One possible solution is the use of drought-inducible promoters and/or tissue specific promoters. Our toolbox includes several native promoters than direct drought-inducible expression and our previous work has identified other similar promoters from tobacco that may also function well in soybean, notably *NtWRKY69*, *NtUPLL2*, and *NtGolS* [16]. Our MEME analyses have shown that the ABRE, DRE and novel GTGC elements are found in the promoters of the most strongly drought-induced genes (Fig. 8). These three elements can form the building blocks for improved synthetic drought-inducible promoters that can be engineered to be paired with transgenes to produce improved expression cassettes for each transgene and each strategy.

However, the difficulties in improving drought tolerance in plants should not be underestimated because drought tolerance is a complex quantitative and multigenic trait with a significant environmental component [2, 3]. The genetic control of traits associated with tolerance to drought often shows low heritability and as a result water stress responses from hydroponics, growth chambers, greenhouses, and field conditions often vary. For this reason the only real judge of success is field performance. Over the years, one of the major problems with transgenic plant lines is that they are ill-defined, neglect physiology and that the phenotypes are unspecific in their definitions. However, a more exact characterization and comparison of transgenic lines can be provided by new advances in phenomics. High-throughput phenotyping will greatly facilitate the characterization of transgenic lines, especially under field conditions, and this precision phenotyping approach should be a major part of strategies to improve drought tolerance (Fig. 10).

### Coumestrol and a possible drought tolerance mechanism

One of the greatest challenges facing agriculture is the availability of water. Any new mechanism that promises to lead to new biotechnological approaches to reduce the amount of water required to irrigate crops is therefore noteworthy. Our data suggest we may have found just such a mechanism linking drought, coumestrol, and mycorrhiza. Isoflavonoids, such as coumestrol, may function as signals in mycorrhizal interactions with plant roots [29]. Coumestrol accumulates to significant levels in mycorrhizal soybean roots [30] and stimulates growth of hyphae of the arbuscular mycorrhizal fungus *Gigaspora margarita* [31]. Coumestrol has also been shown to double the degree of mycorrhizal colonization when added to the soil of mycorrhizal soybean plants [32]. Importantly, mycorrhizal symbiosis can enhance plant tolerance to drought stress through altering plant physiology and gene expression [33]. Under drought stress, mycorrhiza affects water movement into the plant, influencing plant hydration and physiological processes [34]. As a result, mycorrhizal plants can have higher water use efficiency and enhanced growth when irrigation is restored [33].

Our work has provided new information, namely that drought stressed soybean plants very rapidly accumulate coumestrol in the roots. We therefore hypothesize that drought induces a large increase in coumestrol in the roots of legumes. This increase is an inducible mechanism to improve water use efficiency by promoting the growth of mycorrhizal fungi and thereby increasing the amount of water that the plant can reach and/or retain. Coumestrol therefore represents a new target to improve drought tolerance in legumes. However, the enzymes responsible for coumestrol biosynthesis between daidzein and coumestrol are unknown and what little is known comes from tracer studies from the 1970s [35, 36]. Identification of these enzymes is now a priority as they will be required for successful manipulation of coumestrol levels *in planta*.

Unanswered questions include the effect of coumestrol on mycorrhiza. We do not know whether increased mycorrhizal growth is limited to pre-existing mycorrhizal interactions or whether coumestrol promotes new interactions or both. It is also unclear whether the increase in coumestrol levels is the result of *de novo* synthesis or the release of free coumestrol from pools in the vacuole of stored conjugated forms. It has been proposed that an isoflavone conjugate-hydrolyzing β-glucosidase (GmICHG) releases these conjugated isoflavones from their latent forms in the vacuole to be excreted from the roots to promote plant-microbe interactions [37]. Interestingly, our transcriptome analyses reveal that *GmICHG* (*GLYMA12g05770*) is among the 170 most highly expressed genes in soybean roots.

### Drought induced changes in the stomatal development program

It is clear from our data that stomata are a major target for both short and long term responses to drought. Stomatal closure is one of the most rapid responses to drought starting within 30 min and being essentially complete within two hours. A rapid response with similar kinetics is also seen at the mRNA level with the down-regulation of the *GHR1* gene that mediates ABA and hydrogen peroxide-regulated stomatal movement under drought stress. Stomata are also the target of long-term responses to drought stress with fewer stomata on leaves formed after drought (Table 2). Three orthologues of stomatal development genes are among the 29 most highly down-regulated genes in soybean leaves after three hours of drought. One of these genes encodes STOMAGEN an intracellular signaling peptide that is a positive regulator of stomatal patterning. The other two are FAMA/MUTE/SPEECHLESS-like bHLH transcription factors. This agrees with previous research in Arabidopsis [38] and poplar [39]. The situation in soybean is more complex than Arabidopsis even taking into account the ploidy. In several instances, not all paralogs show a similar expression pattern. Also in soybean, the FAMA orthologues are not differentially regulated. Instead, two other more distant members of the clade that are related to all three bHLH genes are very strongly downregulated. The situation in soybean becomes even more complex with the inclusion of six other subfamily 10 (Ia) bHLH genes that form a broader clade with the FAMA/MUTE/SPEECHLESS-like genes. Six of these subfamily 10 (Ia) bHLH genes show strong down-regulation in leaf tissue. One strategy to improving soybean drought tolerance may be to target stomatal density via the manipulation of these genes.

## Conclusions

We have identified targets for the biotechnological improvement of drought responses in soybean. Together with the promoters and promoter elements identified in this study, they form a toolbox of components for strategies to improve drought tolerance. Figure 10 shows how projects using this toolbox could generate improved soybean plants. Precision phenotyping, especially field phenotyping, is an important later component to help determine the exact phenotype of generated plants and the effects of transgene expression on yield, growth, and drought tolerance.

## Methods

### Plant materials

We have recently published an accompanying publication providing a detailed protocol of how we performed the experiments in this report [40]. Briefly, soybean Williams-82 seeds were grown in hydroponics using 0.5× Hoagland solution, pH 5.8 in a growth chamber with a 16 h/8 h day/night cycle at 25 °C and 50 % relative humidity. After 30 days, plants were subjected to water stress by removing them without touching the plants. Leaves and roots were harvested by flash freezing in liquid nitrogen. Nine plants were utilized for each time-point (three replicates per time-point and three plants per replicate). These samples were utilized for all transcriptomics, proteomics and metabolomics experiments.

### Physiological measurements

For TWC (%), three punches of the same diameter were taken and weighed to determine the fresh weight (FW). Samples were lyophilized and dry weight determined (DW). TWC (%) was calculated by (FW-DW)/FW ×100. For osmotic potential, tissues were harvested and frozen at −80 °C in 1.5 ml eppendorf tubes containing a separator and centrifuged for 10 min at 5000 rpm. 10 μl of liquid was used for measuring osmolality (mMol/kg) using an osmometer. Stomatal conductance (mMol/m^2^s) was measured with a steady state diffusion porometer. Phytohormone analysis was performed at the Proteomics and Mass Spectrometry Facility, Danforth Plant Science Center, St Louis, MO. Stomatal density was determined using the impression method. The harvested leaves were covered with clear nail varnish between two auxiliary veins from the central vein to the leaf edge on the abaxial side. A photomicroscope system was used for counting of stomata (s) and epidermal cells (c). Stomatal density was determined as both a function of leaf surface area and as leaf stomatal index [s/(s + c)] × 100] [41]. 80 clear varnish stomatal imprints were collected from 26 different leaves which were harvested from 14 separate drought treated plants. 102 imprints were taken from 37 leaves which were harvested from 17 non-drought plants.

### Transcriptomics analyses

RNA was isolated using QIAGEN© RNeasy-MIDI. 10 μg total RNA from each sample was used for micro-array analysis. A custom made 12 × plex array was designed by Roche NimbleGen, Inc. containing multiple 60mer oligomers to all genes from the GLYMAv1.0 release of the soybean genome. Oligoarray experiments were performed at MOgene, LLC (St Louis, MO). Data analysis was performed using ArrayStar v4. Differential regulation was calculated using 90 % confidence (FDR Benjamini Hochberg) and 8-fold change. For gene enrichment analysis, agriGO [42] was employed and enriched GO terms were obtained using Singular Enrichment Analysis [43]. Pathway visualization was performed by MapMan. The transcriptome data set is available in the Gene Expression Omnibus under the accession number GSE49537.

### Motif analyses

Conserved motifs in promoters were found using MEME (http://meme-suite.org/tools/meme) [24] with motif widths set at eight nucleotides as described in Tripathi et al. [44].

### Proteomics analyses

Roots tissues were processed at Bio-Proximity, LLC as described [45, 46]. MGF data files were searched using X!Hunter against the latest library on the GPM [47] and also searched using X!Tandem [48, 49] using both the native and k-score [50] scoring algorithms and by OMSSA [51]. Proteins were required to have 2 or more unique peptides with E-value scores of 0.01 or less. The proteomics data was used for identification of differentially regulated proteins with an FDR correction of 5 %.

### Metabolomics analyses

Metabolomics analyses were performed at Metabolon, Inc. (North Carolina). The global unbiased metabolic profiling platform was based on a combination of three independent platforms: UHLC/MS/MS2 optimized for basic species, UHLC/MS/MS2 optimized for acidic species, and GC/MS. This platform has been described in detail [52]. Three replicates were used per time-point and rigorous statistical analyses were performed. Following log transformation and imputation with minimum observed values for each compound, Welch’s two-sample t-test was used to identify biochemicals that differed significantly between different time points and in different tissues. The statistical significance threshold was set at *p* ≤ 0.05. An estimate of the false discovery rate (q-value) was also calculated (Additional file 1: Table S1) to take into account the multiple comparisons in the study and a low q-value (q < 0.10) showed an indication of high confidence in the major results.

### Soybean hairy-root transformation and GFP Quantification

Promoter sequences (1 kb upstream from the ATG) including the 5′UTRs were obtained from phytozome [53]. The promoters were cloned into pFLEV [54] and transformed into LBA4404 agrobacterium cells by electroporation.

Soybean hairy-root transformation was performed as described [55]. After 3–4 weeks, the plants were transferred to hydroponics and dehydration was performed as described above. The roots were observed under an OLYMPUS AX70 upright compound microscope. Eleven to fourteen transformed hairy-roots were analyzed per construct. GFP quantification was performed with Image J [56].

For measuring ABA inducibility, transformed roots were placed in 20 μM ABA for 24 h. For cold treatment, plants were transferred to boxes with ice. For salt treatment, plants were placed in 150 mM NaCl for 24 h.

## Availability of data and materials

We have made the soybean oligo array data available at the Gene Expression Omnibus online repository as GEO accession GSE49537.

## Additional files

Additional file 1: Table S1. Heat maps and box plots of all detectable metabolites of known identity. (XLSX 1476 kb) Additional file 2: Figure S1. Up regulation of raffinose and galactinol synthase genes correlates with increases in galactinol and raffinose. (PDF 236 kb) Additional file 3: Figure S2. Metabolite analysis of the ammonia detoxification processes (PDF 184 kb) Additional file 4: Table S2. Complete list of differentially expressed genes at the mRNA level at the different time points in leaves and roots (XLSX 1382 kb) Additional file 5: Figure S3. Gene ontology analysis. (PDF 315 kb) Additional file 6: Table S3. Significant GO terms root 1 h. (XLSX 12 kb) Additional file 7: Table S4. Significant GO terms root 30 min. Molecular Function and Biological Process category enriched GO terms. (XLSX 14 kb) Additional file 8: Table S5. Significant GO terms root 5 h. (XLSX 18 kb) Additional file 9: Table S6. Significant GO terms leaf 2 h. (XLSX 14 kb) Additional file 10: Table S7. Significant GO terms leaf 5 h. (XLSX 17 kb) Additional file 11: Table S8. List of differentially expressed TF genes after 30 min in roots (XLSX 12 kb) Additional file 12: Table S9. List of differentially expressed TF genes after 2 h in leaves (XLSX 23 kb) Additional file 13: Table S10. Differentially expressed calcium signaling-associated genes from MapMan (XLSX 16 kb) Additional file 14: Table S11. bHLH genes in soybean. (XLSX 18 kb) Additional file 15: Figure S4. Phylogenetic analyses of the bHLH transcription factor family from soybean. (PDF 71 kb) Additional file 16: Table S12. Soybean orthologs of Arabidopsis stomatal development genes. (DOC 34 kb) Additional file 17: Table S13. qRT-PCR verification of expression patterns of 14 genes from the oligo array analysis. (PDF 1550 kb) Additional file 18: Table S14. Differentially accumulated proteins during dehydration in soybean roots. (XLSX 23 kb)
