# Supplementary material for: A toolbox of genes, proteins, metabolites and promoters for improving drought tolerance in soybean includes the metabolite coumestrol and stomatal development genes
Source: BMC Genomics. 2016 Feb 9;17:102. doi: 10.1186/s12864-016-2420-0 (PMC4746818; doi:10.1186/s12864-016-2420-0)
Supplement: Additional file 2: Figure S1. — Up regulation of raffinose and galactinol synthase genes correlates with increases in galactinol and raffinose. (PDF 236 kb) [file 12864_2016_2420_MOESM2_ESM.pdf]

a.

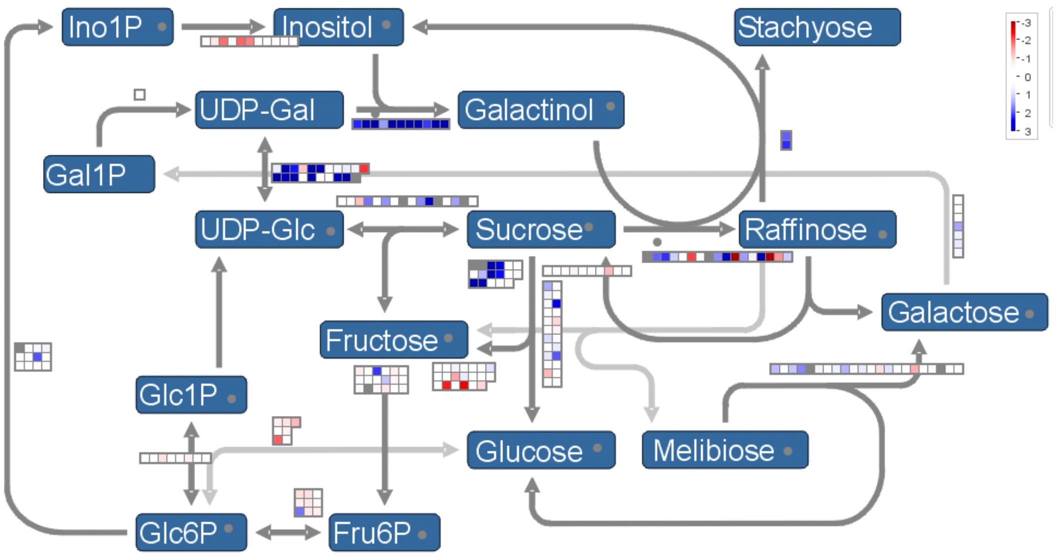
Ino1P
Inositol
Stachyose
UDP-Gal
Galactinol
Gal1P
UDP-Glc
Sucrose
Raffinose
Galactose
Fructose
Glc1P
Glucose
Melibiose
Glc6P
Fru6P
-3
-2
-1
0
1
2
3

b.

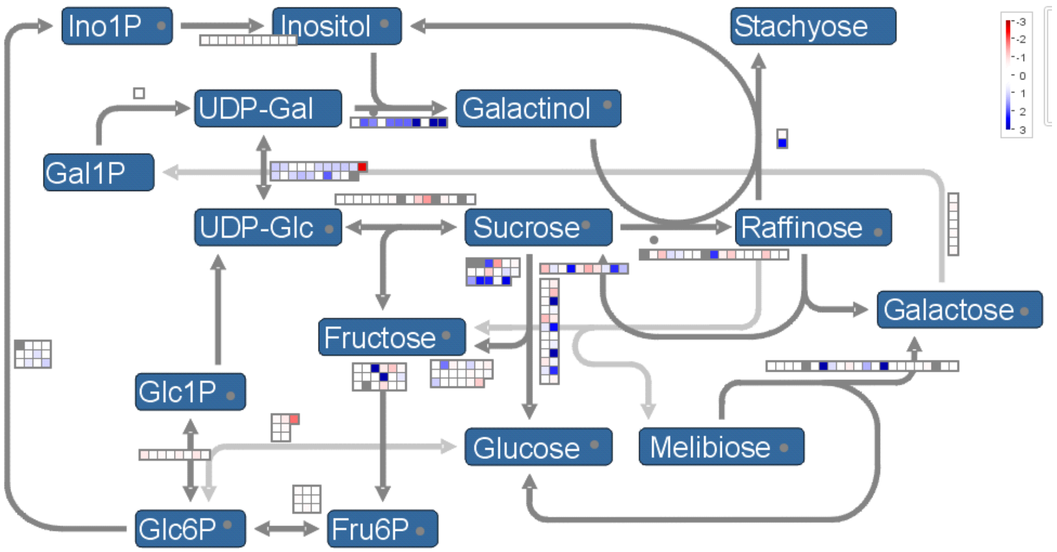
Ino1P
Inositol
Stachyose
UDP-Gal
Galactinol
Gal1P
UDP-Glc
Sucrose
Raffinose
Galactose
Fructose
Glc1P
Glucose
Melibiose
Glc6P
Fru6P
-3
-2
-1
0
1
2
3

c.

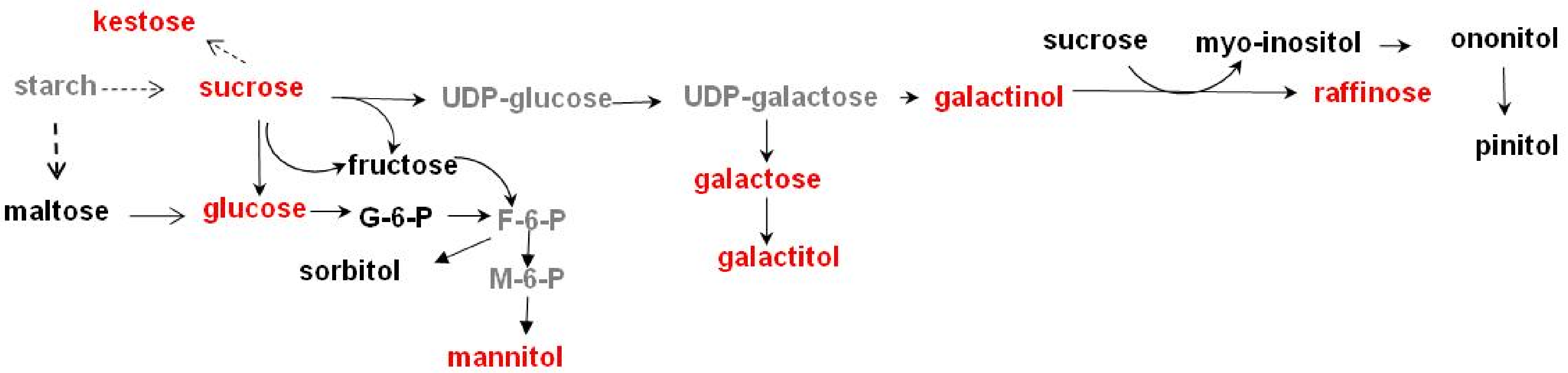

d.

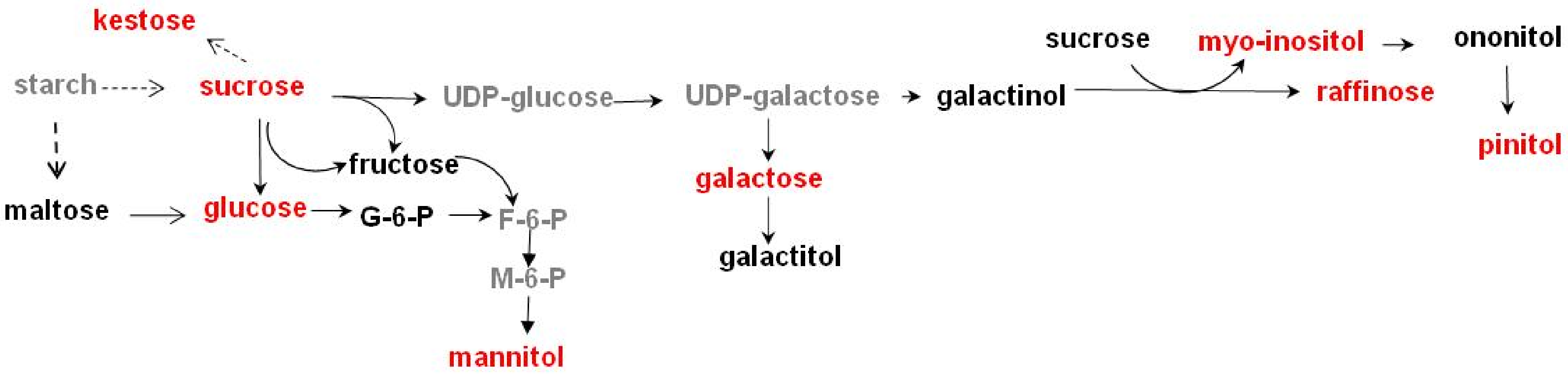

Figure S1. Up regulation of raffinose and galactinol synthase genes correlates with increases in galactinol and raffinose. MapMan visualization of differentially expressed genes in the galactinol and raffinose biosynthetic pathways in (a) leaves and (b) roots. The figure was constructed using log2-transformed ratios of induced versus control. The most intense colors represent 8-fold change. Blue denotes increase and red decrease. Overviews of metabolite accumulation in (c) leaves and (d) roots. Red indicates a statistically significance ($p \leq 0.05$) increase in sugar level as determined by Welch's two-sample t-test.
