# Supplementary material for: A toolbox of genes, proteins, metabolites and promoters for improving drought tolerance in soybean includes the metabolite coumestrol and stomatal development genes
Source: BMC Genomics. 2016 Feb 9;17:102. doi: 10.1186/s12864-016-2420-0 (PMC4746818; doi:10.1186/s12864-016-2420-0)
Supplement: Additional file 3: Figure S2. — Metabolite analysis of the ammonia detoxification processes (PDF 184 kb) [file 12864_2016_2420_MOESM3_ESM.pdf]

a.

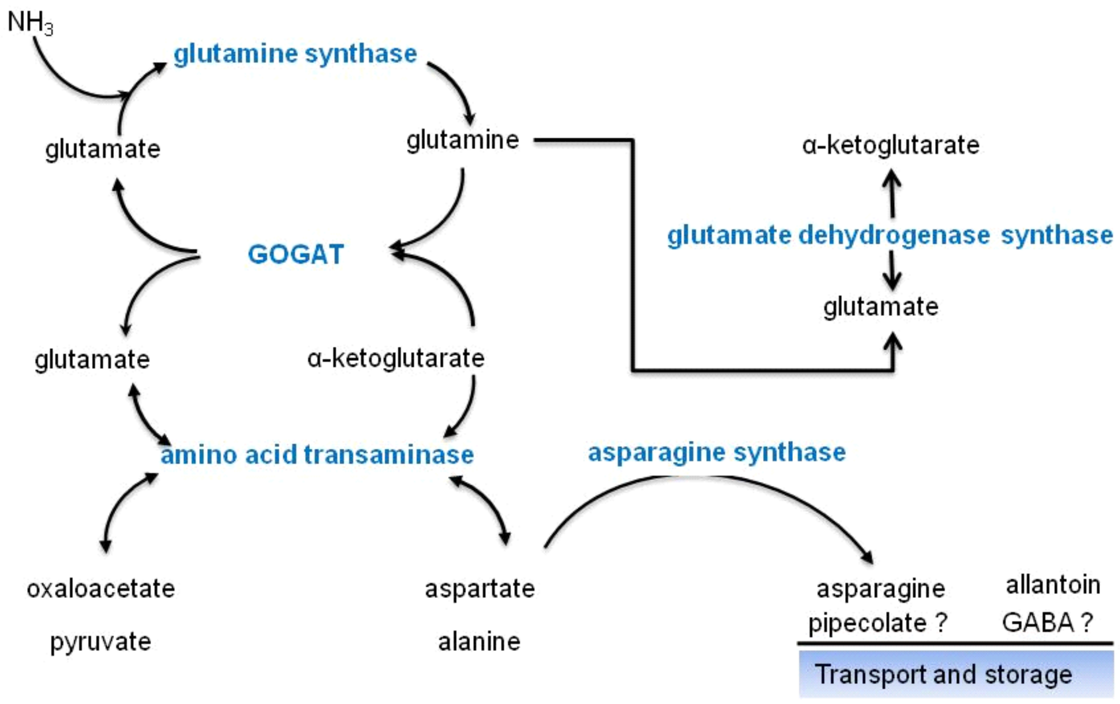
NH_3
glutamine synthase
glutamate
glutamine
GOGAT
glutamate
α-ketoglutarate
amino acid transaminase
oxaloacetate
pyruvate
aspartate
alanine
asparagine synthase
α-ketoglutarate
glutamate dehydrogenase synthase
glutamate
asparagine
pipecolate ?
allantoin
GABA ?
Transport and storage

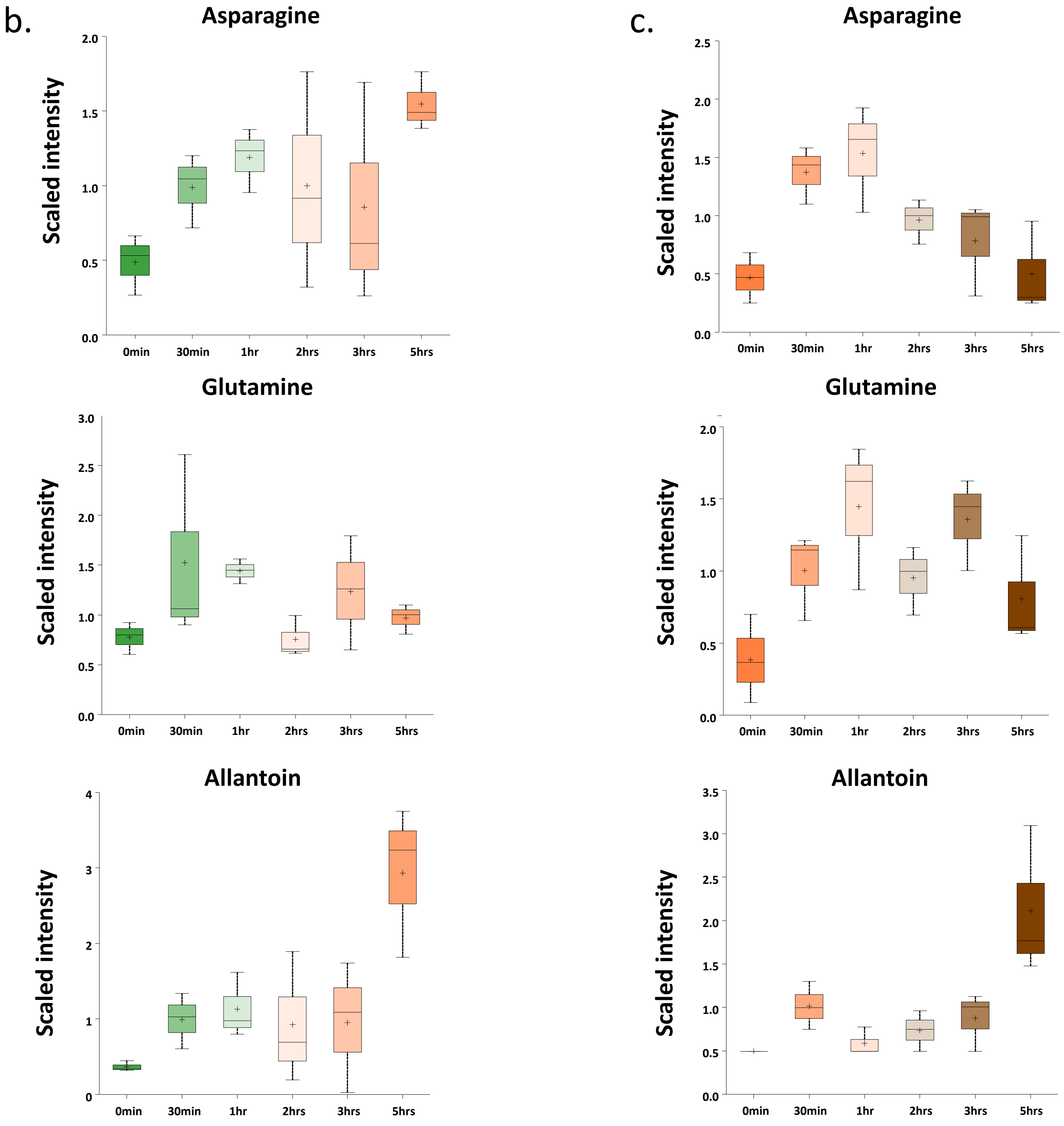
b.
Asparagine
Scaled intensity
0.0
0.5
1.0
1.5
2.0
0min
30min
1hr
2hrs
3hrs
5hrs
Glutamine
Scaled intensity
0.0
0.5
1.0
1.5
2.0
2.5
3.0
0min
30min
1hr
2hrs
3hrs
5hrs
Allantoin
Scaled intensity
0
1
2
3
4
0min
30min
1hr
2hrs
3hrs
5hrs
c.
Asparagine
Scaled intensity
0.0
0.5
1.0
1.5
2.0
2.5
0min
30min
1hr
2hrs
3hrs
5hrs
Glutamine
Scaled intensity
0.0
0.5
1.0
1.5
2.0
0min
30min
1hr
2hrs
3hrs
5hrs
Allantoin
Scaled intensity
0.0
0.5
1.0
1.5
2.0
2.5
3.0
3.5
0min
30min
1hr
2hrs
3hrs
5hrs

d.

Biochemical Name	Fold Change T30/T0	T60/T0	T120/T0	T180/T0	T300/T0
Root					
allantoin	**2.05**	1.19	1.49	1.77	**4.27**
allantoic acid	**2.99**	1.62	1.23	1.76	**2.29**
asparagine	**2.94**	**3.29**	**2.06**	1.68	1.07
glutamine	2.6	3.75	2.47	3.52	2.09
Leaf					
allantoin	**2.69**	**3.07**	2.51	2.59	**7.97**
asparagine	**2.03**	**2.44**	2.05	1.76	**3.17**
glutamine	1.97	**1.86**	0.97	1.59	1.25

Figure S2. Metabolite analysis of the ammonia detoxification processes. (a) Biosynthetic pathways associated with ammonia detoxification. (b) Box plot of asparagine, glutamine, and allantoin levels in leaf (b) and root (c). Crosses indicate the mean value, horizontal lines the median value, shaded boxes indicate the upper and lower quartiles, and the top and bottom of the bars indicate the maximum and minimum of the distribution. (d) Heat map of statistically significant changes in asparagine, glutamine, and allantoin levels. Red cells indicate $p \leq 0.05$ with the mean values significantly higher than the control. Light red cells indicate $0.05 < p < 0.10$.
