# Supplementary material for: A toolbox of genes, proteins, metabolites and promoters for improving drought tolerance in soybean includes the metabolite coumestrol and stomatal development genes
Source: BMC Genomics. 2016 Feb 9;17:102. doi: 10.1186/s12864-016-2420-0 (PMC4746818; doi:10.1186/s12864-016-2420-0)
Supplement: Additional file 5: Figure S3. — Gene ontology analysis. (PDF 315 kb) [file 12864_2016_2420_MOESM5_ESM.pdf]

a.

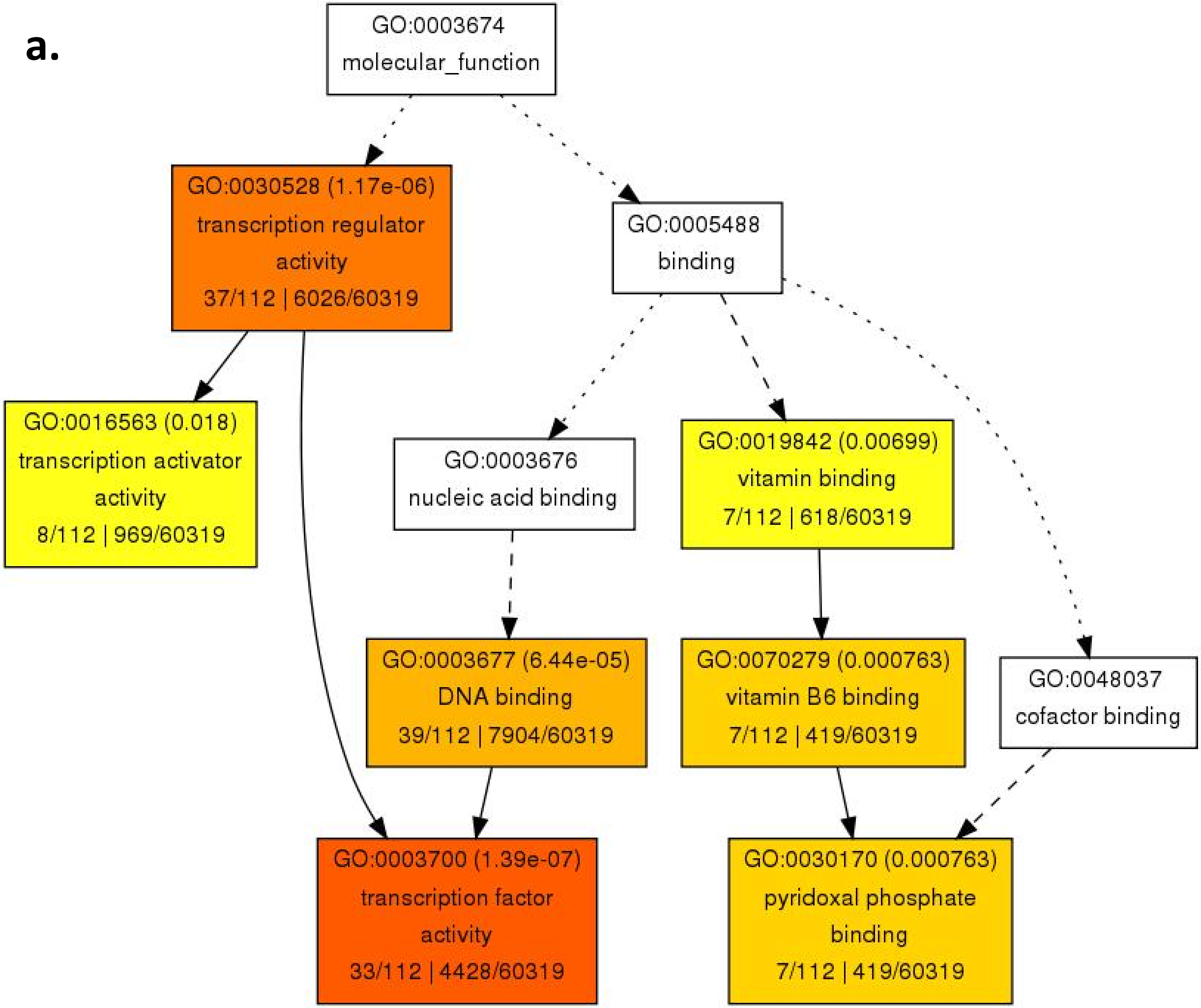
GO:0003674
molecular_function
GO:0030528 (1.17e-06)
transcription regulator activity
37/112 | 6026/60319
GO:0005488
binding
GO:0016563 (0.018)
transcription activator activity
8/112 | 969/60319
GO:0003676
nucleic acid binding
GO:0019842 (0.00699)
vitamin binding
7/112 | 618/60319
GO:0003677 (6.44e-05)
DNA binding
39/112 | 7904/60319
GO:0070279 (0.000763)
vitamin B6 binding
7/112 | 419/60319
GO:0048037
cofactor binding
GO:0003700 (1.39e-07)
transcription factor activity
33/112 | 4428/60319
GO:0030170 (0.000763)
pyridoxal phosphate binding
7/112 | 419/60319

b.

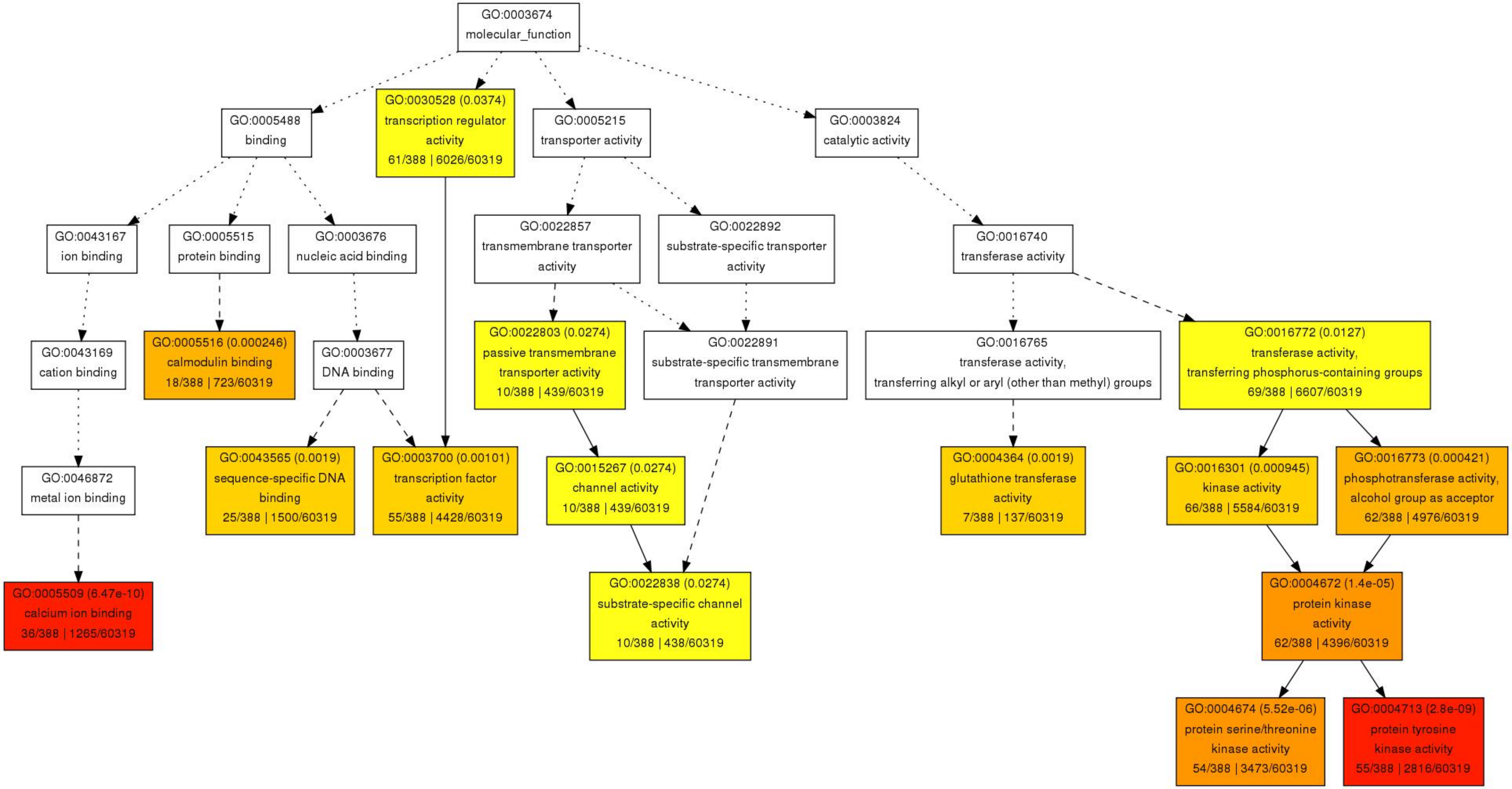

Figure S3. Gene ontology analysis. (a) Molecular Function category enriched GO terms in roots after 30 minutes of water stress. (b) Molecular Function category enriched GO terms in leaf after two hours of water stress. agriGO was employed for analysis and enriched GO terms were obtained using Singular Enrichment Analysis . Colors reflect the level of significance.
