# Supplementary material for: A toolbox of genes, proteins, metabolites and promoters for improving drought tolerance in soybean includes the metabolite coumestrol and stomatal development genes
Source: BMC Genomics. 2016 Feb 9;17:102. doi: 10.1186/s12864-016-2420-0 (PMC4746818; doi:10.1186/s12864-016-2420-0)
Supplement: Additional file 15: Figure S4. — Phylogenetic analyses of the bHLH transcription factor family from soybean. (PDF 71 kb) [file 12864_2016_2420_MOESM15_ESM.pdf]

a.

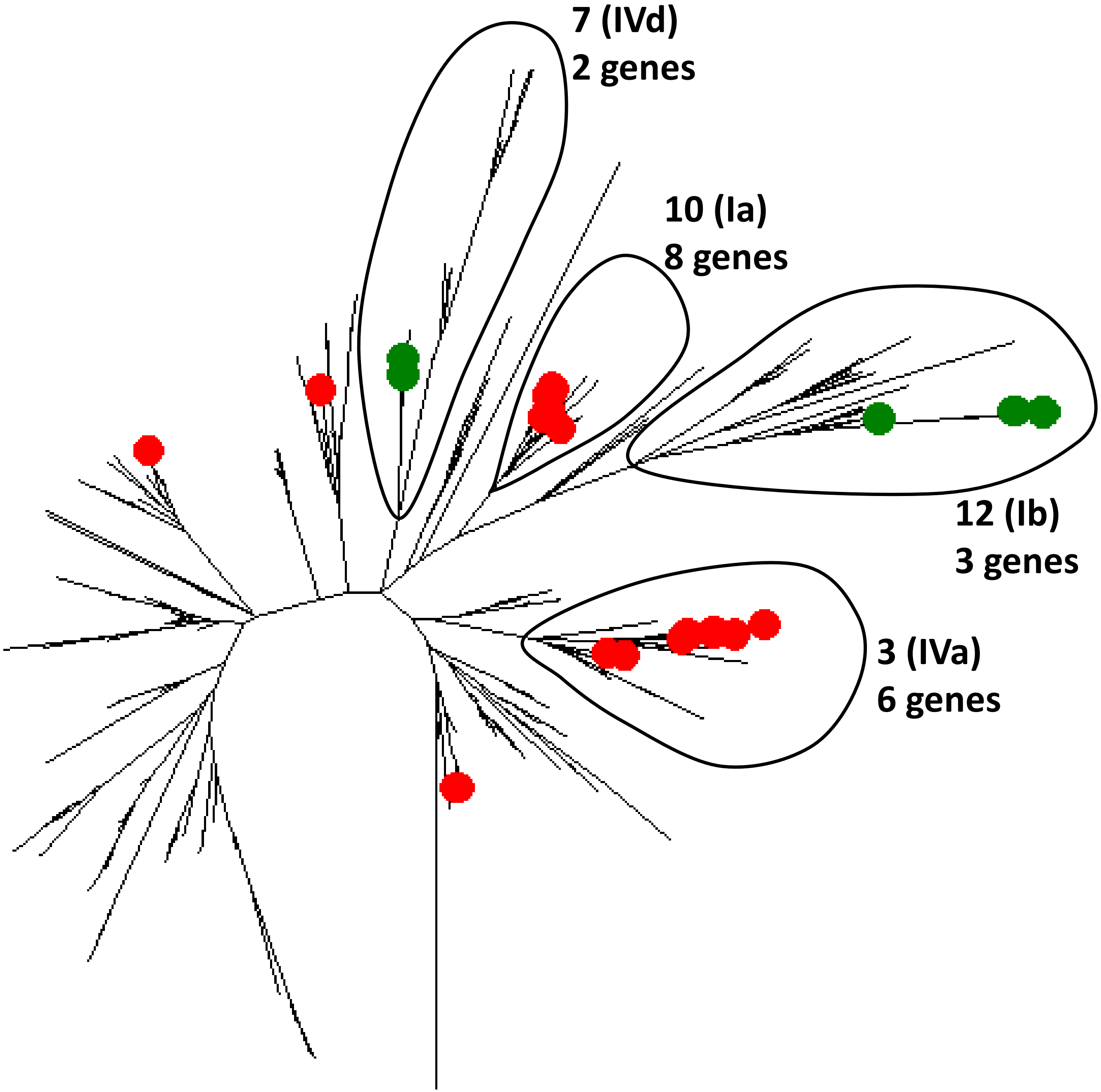

b.

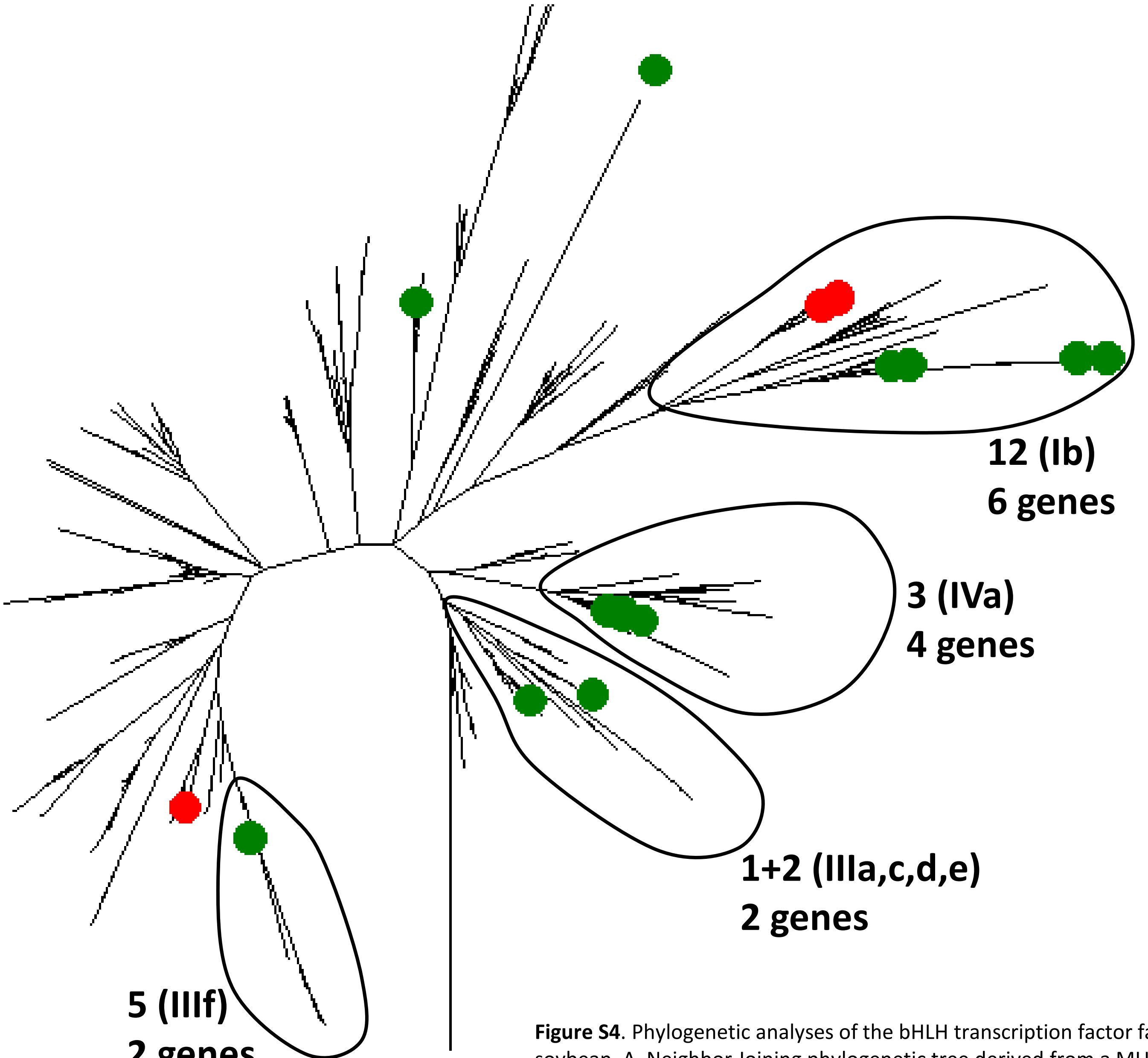

Figure S4. Phylogenetic analyses of the bHLH transcription factor family from soybean. A. Neighbor Joining phylogenetic tree derived from a MUSCLE alignment of the full length proteins. Red dots denote 8-fold down-regulation and green dots 8-fold up-regulation in roots. B. The same phylogenetic tree as shown in A. but with the inducibilities in leaf.
