# Supplementary material for: A toolbox of genes, proteins, metabolites and promoters for improving drought tolerance in soybean includes the metabolite coumestrol and stomatal development genes
Source: BMC Genomics. 2016 Feb 9;17:102. doi: 10.1186/s12864-016-2420-0 (PMC4746818; doi:10.1186/s12864-016-2420-0)
Supplement: Additional file 17: Table S13. — qRT-PCR verification of expression patterns of 14 genes from the oligo array analysis. (PDF 1550 kb) [file 12864_2016_2420_MOESM17_ESM.pdf]

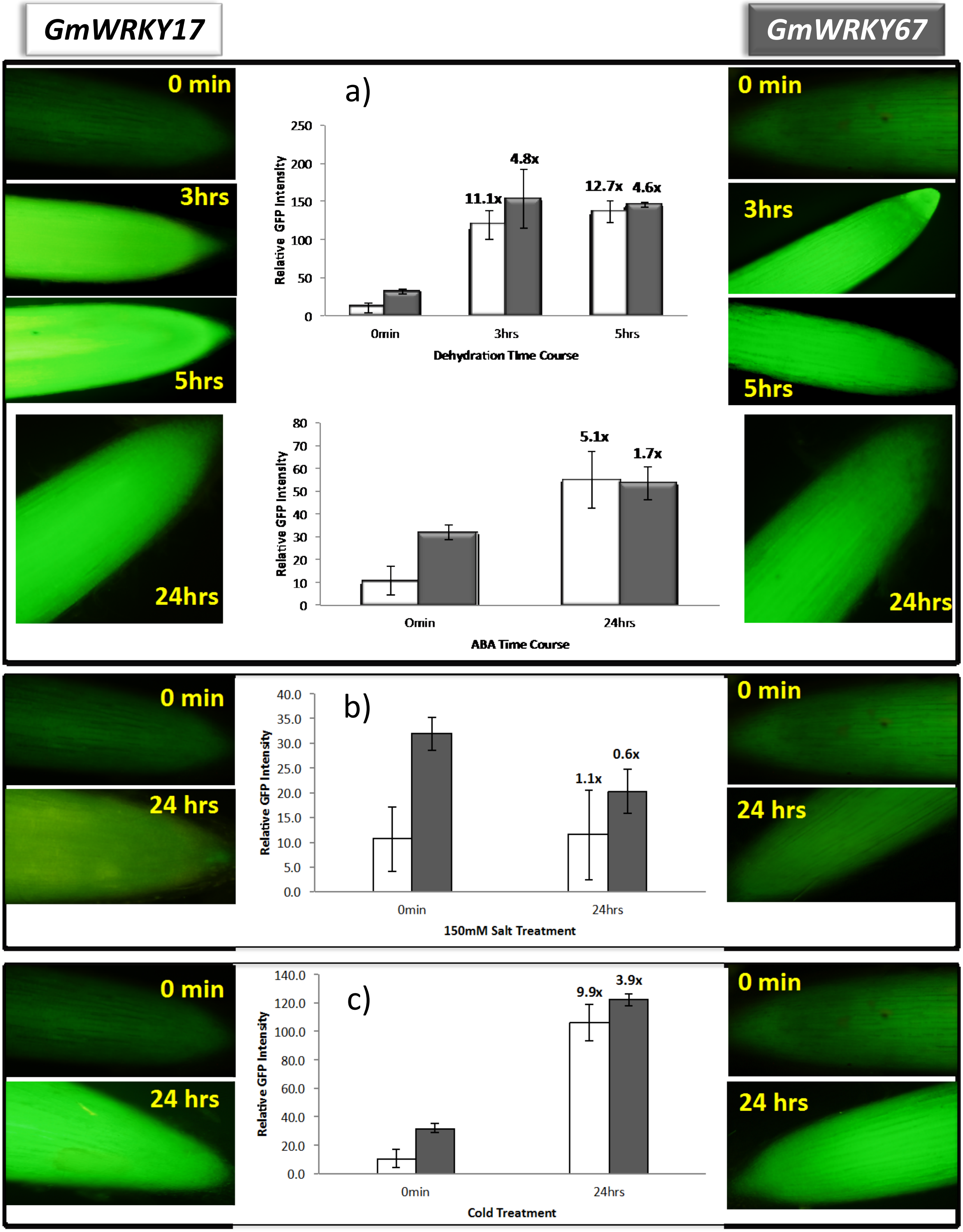

Figure 9. Validation of promoter activity of *GmWRKY17* and *GmWRKY67* in soybean hairy-roots during dehydration and 20µM ABA treatment. Validation of promoter activity via visualization and quantification of a promoter:GFP construct of *GmWRKY17* and *GmWRKY67* in soybean hairy-roots during (a) dehydration (b) 20µM ABA treatment and (c) cold. The time points in hours are shown. The graph shows mean ± standard error for 9 independent plants for each time point. The fold inducibilities are indicated.
